# An organ-derived extracellular matrix triggers in situ kidney regeneration in a preclinical model

**DOI:** 10.1038/s41536-022-00213-y

**Published:** 2022-02-28

**Authors:** Kazuki Tajima, Hiroshi Yagi, Toshinori Morisaku, Kotaro Nishi, Hiroko Kushige, Hideaki Kojima, Hisanobu Higashi, Kohei Kuroda, Minoru Kitago, Shungo Adachi, Tohru Natsume, Kumiko Nishimura, Mototsugu Oya, Yuko Kitagawa

**Affiliations:** 1 Department of Surgery, Keio University School of Medicine, Shinanomachi 35, Shinjuku, Tokyo, Japan; 2 Department of Small Animal Internal Medicine, Kitasato University School of Veterinary Medicine, Higashi 23–35–1, Towada, Aomori, Japan; 3 Cellular and Molecular Biotechnology Research Institute, National Institute of Advanced Industrial Science and Technology (AIST), Aomi 2–4–7, Koto, Tokyo, Japan; 4 Department of Urology, Keio University School of Medicine, Shinanomachi 35, Shinjuku, Tokyo, Japan

**Keywords:** Translational research, Regenerative medicine, Biomedical materials, Tissue engineering, Preclinical research

## Abstract

It has not been considered that nephrons regenerate in adult mammals. We present that an organ-derived extracellular matrix in situ induces nephron regeneration in a preclinical model. A porcine kidney-derived extracellular matrix was sutured onto the surface of partial nephrectomy (PN)-treated kidney. Twenty-eight days after implantation, glomeruli, vessels, and renal tubules, characteristic of nephrons, were histologically observed within the matrix. No fibrillogenesis was observed in the matrix nor the matrix-sutured kidney, although this occurred in a PN kidney without the matrix, indicating the structures were newly induced by the matrix. The expression of renal progenitor markers, including Sall1, Six2, and WT-1, within the matrix supported the induction of nephron regeneration by the matrix. Furthermore, active blood flow was observed inside the matrix using computed tomography. The matrix provides structural and functional foundations for the development of cell-free scaffolds with a remarkably low risk of immune rejection and cancerization.

## Introduction

The human kidney is comprised of ~750,000–2,000,000 nephrons, which are a critical functional unit in the kidney^1,2^. Since adult mammals exhibit a limited capacity for nephron regeneration, it has not been observed that kidney functions are recovered in patients with chronic renal failure^3,4^. Kidney failure is defined as the loss of nephron functions, such as reduced glomerular filtration rate and increased urinary albumin excretion^5^. Thus, such chronic kidney diseases progress to end-stage renal diseases (ESRD). Currently, available treatment options for ESRD are limited to dialysis and transplantation. While dialysis may compensate for impaired renal functions, it involves extracorporeal circulation, which entails risks of thrombosis and infection^5^. In addition, since dialysis creates a financial burden for many patients, it may not be economically feasible^5^. Therefore, dialysis does not ensure the long-term survival of patients with ESRD^6^. Although renal transplantation is curative for individuals with ESRD, the number of recipients awaiting transplantation exceeds the availability of organ donors. Moreover, acute graft rejection and failure have not been overcome by transplantation. Thus, the development of transplantable kidney and nephron regeneration technologies is required. The successful implementation of these approaches will help to reduce medical costs and improve prognosis, productivity, and quality of life for patients with renal diseases.

Recent advances in bioengineering have established an organ regeneration-based infrastructure as an alternative to organ transplantation. Decellularization has been explored as a powerful technique for the reconstruction of three-dimensional organ structures. In this technique, all living cells from organs or tissues are removed, leading to the conservation of organ-derived translucent three-dimensional native matrices. The native matrices contain not only organ-specific architecture, such as extracellular matrices (ECMs) and vascular networks, but also signaling molecules, such as growth factors and cytokines. These features provide signals for vital functions, including cellular adhesion, migration, and differentiation and gene expression in the matrices^7–12^. Thus, organ-derived native matrices offer structural support and ideal fields for organ regeneration. Previously, we reported the efficacy of liver and islet-derived native matrices as regenerative organs and demonstrated that the matrices support cellular functions and viability^12–15^.

Some in vitro studies have demonstrated the efficacy of kidney-derived extracellular matrices in kidney wound repair and regeneration. Song *et al*. observed the regeneration of functional glomeruli with a rat kidney-derived bio-scaffold seeded with epithelial and endothelial cells and the resultant production of rudimentary urine^7^. Further, it was reported that embryonic stem cells proliferate and differentiate within rat kidney-derived bio-scaffolds^16,17^. Conversely, in vivo studies on kidney-derived extracellular matrix have been limited to the use of rat models and there is no report on the implantation of the matrix in clinical and preclinical models using large animals^7^. In addition, after its recellularization, the matrix was anastomosed to the renal artery and vein of a rat^7^. Although the implantation of the recellularized matrix provided excretory functions in rats, in general, large amounts of cells are needed for the recellularization of organ-derived native matrices. Immune responses of recipients to the implanted cells, especially pluripotent cells, such as embryonic stem cells and induced pluripotent stem cells, and cancerization induced by such cells are also a concern. Thus, as a probable method for the realization of safe and low-cost organ regeneration with organ-derived native matrices, it is highly desired that the matrices are directly implanted to recipients without cell seeding. The method is based on the induction of structural and functional wound repair and regeneration of damaged organs due to native matrix-assisted self-repair. Furthermore, it largely contributes to the regeneration of not only impaired kidneys due to chronic renal failure but also kidneys with partial nephrectomy (PN)-applied disorders, such as small-sized kidney cancer.

The present study aimed to investigate the ability of a kidney-derived extracellular matrix for renal regeneration in a preclinical model using a large animal model. In our previous porcine study, an implanted organ-derived extracellular matrix promoted structural reorganization after liver resection^18^. Herein, we implanted a porcine kidney-derived matrix onto the resected surface of a porcine PN kidney to elucidate whether the matrix induced a structural foundation for renal regeneration with active blood flow. The study is the evidence in a preclinical model that an organ-derived extracellular matrix can induce in situ renal regeneration, which provides a platform for further research into treatment options for renal diseases and the development of organ-derived scaffolds with a remarkably low risk of immune rejection and cancerization.

## Results

### Preparation of a decellularized porcine kidney

**Fig. 1:**
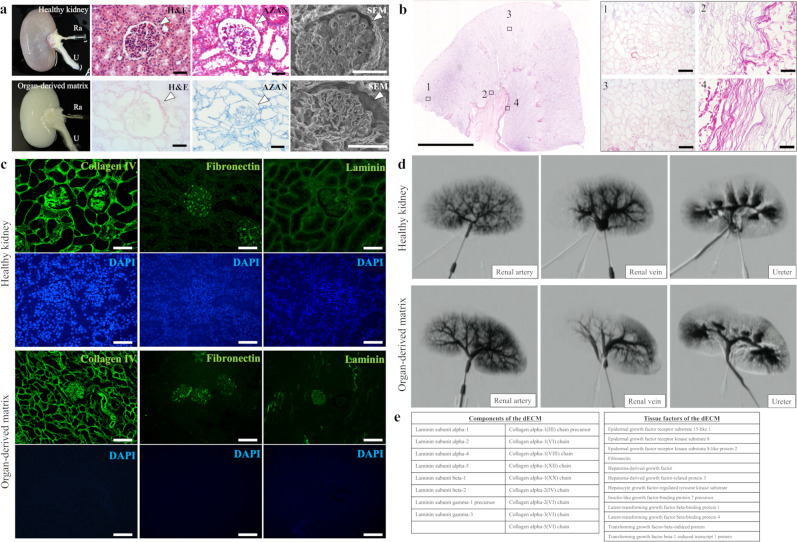
Decellularization of the entire porcine kidney. **a** Macro-scale photographs, H&E and Azan staining images, and SEM images for (top) a healthy kidney and (bottom) the organ-derived matrix. The white arrowheads show glomeruli. Scale bar: 30 μm for H&E and Azan staining images, 50 μm for SEM images. Ra, renal artery; U, ureter. **b** (left) Macro-scale montage image of H&E-stained organ-derived matrix in the whole section and (right) each enlarged region within the black-edged squares in the image. Scale bar: (left) 5 mm, (right) 50 μm. **c** Representative immunohistochemical images of collagen type IV, fibronectin, and laminin staining for a healthy kidney and the organ-derived matrix counterstained with DAPI. Scale bar: 100 μm. **d** Contrast-enhanced fluoroscopic X-ray images of a healthy kidney and the organ-derived matrix. **e** List of residual ECM and growth factor components in the organ-derived matrix obtained via exhaustive protein analysis using LC-MS.

Hematoxylin and eosin (H&E) and Azan staining, scanning electron microscopy (SEM)-imaging, and immunohistochemical analyses were performed to verify the feasibility for the decellularization of a porcine kidney based on our protocol. A harvested porcine kidney was decellularized by perfusion with sodium dodecyl sulfate (SDS) through a cannulated renal artery. The histological and SEM images showed that the cytoplasmic and nuclear components were removed, and the cellular framework was preserved including the whole kidney’s microvascular structures (Fig. 1a, Supplementary Fig. 1). The whole sectional images of the decellularized kidney also showed no survival of cellular components (Fig. 1b). DAPI staining demonstrated the absence of positive cells in the kidney (Supplementary Fig. 2). These data showed the absence of any residual cells in the kidney. Compared with an intact kidney, the decellularized kidney retained functional ECM components, such as collagen type IV, fibronectin, and laminin, according to immunohistochemical analyses (Fig. 1c). The vascular robustness was also maintained in the kidney (Fig. 1d). In addition, the exhaustive protein analysis revealed the survival of ECM components and signaling molecules in the kidney (Fig. 1e, Supplementary Tables 1 and 2). The prepared decellularized kidney preserved the organ-specific architecture, including ECMs and vascular networks, and incorporated an array of signaling molecules.

### Preparation of the PN model (control) and the organ-derived matrix-sutured model

**Fig. 2:**
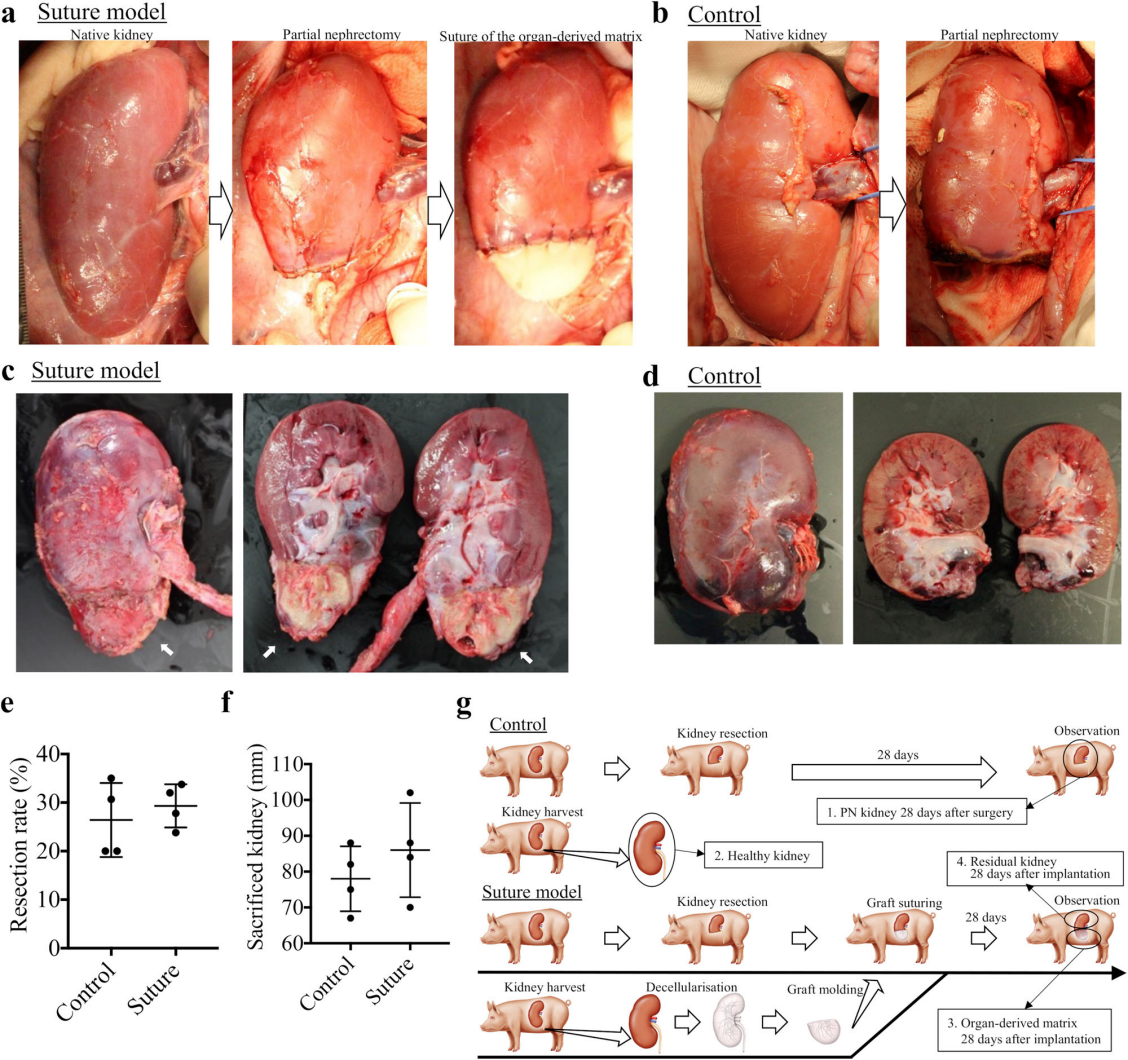
Design of the porcine partial nephrectomy (PN) (control) and the suture model of the organ-derived matrix. **a** Scheme for the construction of the matrix-sutured model. A porcine native kidney (left) is subjected to PN (middle), then the organ-derived matrix is sutured onto the resected surface of the native kidney (right). **b** Scheme for the construction of the control model. A porcine native kidney is only subjected to PN. **c** Photographs of a harvested kidney containing an organ-derived matrix sutured to residual kidney 28 days postimplantation. The white arrows show the implanted organ-derived matrix. **d** Photographs of a harvested PN kidney 28 days post-surgery. **c**, **d** The images on the right show the sagittal section of the harvested kidneys. **e** Comparison of the resection rate between the PN kidney (control) and the matrix-sutured kidney. Bars represent the mean ± SD, (*n* = 4). **f** Comparison of the major axes between the harvested PN kidney (control) and matrix-sutured kidney. Bars represent the mean ± SD, (*n* = 4). **g** Experimental outline of the porcine PN model (control) and the matrix-sutured model. The following four types of harvested kidneys were analyzed: PN kidney 28 days postsurgery (control, sample 1), healthy kidney (sample 2), organ-derived matrix 28 days post-implantation (sample 3), and corresponding residual kidney 28 days post-implantation (sample 4).

A pig was subjected to PN according to a previously reported method with a slight modification^19^. Briefly, after aseptic laparotomy, the peritoneum overlying the porcine kidney was medically incised. About one-third of each caudal kidney was excised with a saline-based bipolar system. The sterile acellular organ-derived matrix was molded under aseptic conditions, then sutured onto the PN kidney surface through contact with its renal parenchyma (Fig. 2a). As a control, samples subjected to PN were prepared (Fig. 2b). Twenty-eight days post-surgery, the pigs with the matrix-sutured kidney or the control were sacrificed, and each kidney was harvested (samples 1, 3, and 4 in Fig. 2g). From macroscopic observations, the morphology of the organ-derived matrix was well-preserved, and no sign of rejection or absorption was observed (Fig. 2c). The matrix adhered well to the incised surface of the native kidney. In contrast, the resected kidney in the control did not regenerate as expected (Fig. 2d)^3,4^. The resection rate of each kidney was similar (Fig. 2e) compared with the control and the matrix-sutured model, while the PN kidney in the control showed slight atrophy after the surgery (Fig. 2f).

### Induction of wound repair unaccompanied by fibrillogenesis due to the organ-derived matrix

**Fig. 3:**
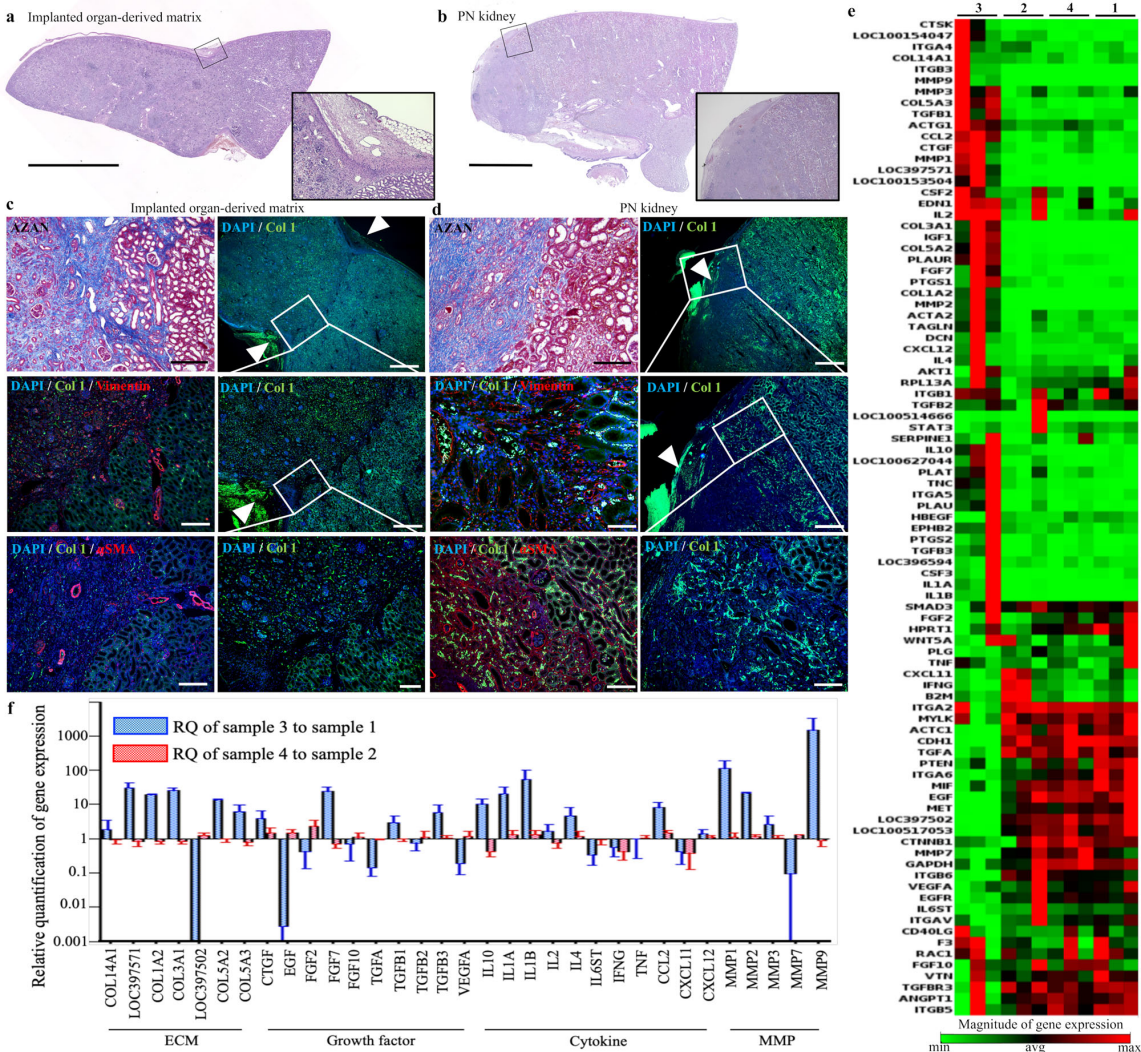
The organ-derived matrix-induced wound repair unaccompanied by fibrillogenesis. **a**, **b** Macro-scale montage images of H&E staining in **a** the organ-derived matrix sutured to the residual kidney and **b** the PN kidney. The square insets show the magnification of the regions surrounded by black-edged squares in the images. Scale bar: 5 mm. **c**, **d** Histological images of Azan staining **c** around the boundary between the residual kidney and the organ-derived matrix and **d** near the resection line in the PN kidney, and immunohistochemical images of collagen type I/vimentin, collagen type I/αSMA, and collagen type I staining in the regions counterstained with DAPI. The images of collagen type I staining at varied magnifications are shown, indicating the appearance of the boundary and the resection area. The white arrowheads show **c** the boundary or **d** the point where the discontinuity of the kidney capsule was observed. Scale bar: 200 μm for Azan staining, 200 μm for collagen type I/vimentin or αSMA staining, and 1 mm, 500 μm, or 200 μm for collagen type I staining. **e** Clustergram showing the expression of 89 wound repair-related genes in the PN kidney 28 days post-surgery (sample 1), the healthy kidney (sample 2), the organ-derived matrix 28 days post-implantation (sample 3), and the residual kidney 28 days post-implantation (sample 4). **f** Quantification of expression levels for ECM-, growth factor-, cytokine-, and ECM-remodeling enzyme (MMP)-related genes in Fig. 3e. Blue and red histograms show the relative values of sample 3 to sample 1 and those of sample 4 to sample 2, respectively. Bars represent the mean ± SD, (*n* = 3).

In damaged organs or tissues, wound repair typically proceeds, where cells, such as fibroblast and vascular endothelial cells, migrate into the organs or tissues, followed by the formation of nonfunctioning fibrotic tissues well-known as scars^20^. However, it has been reported that organ-derived materials can promote the non-fibrotic formation of organs or tissues through distinct healing processes from normal repair, including cellular proliferation, angiogenesis, stem/progenitor cell recruitment, and modulated immune system, although the underlying mechanism has not been fully understood^11,21,22^. Thus, the organ-derived matrix-sutured kidney was histologically compared with the control (PN kidney) to examine whether fibrillogenesis occurred in the matrix-sutured kidney. We first confirmed that the boundary between the matrix and the adjoining residual kidney or the resection area in the PN kidney could be distinguished via H&E and Azan staining, as well as collagen type I immunohistochemical imaging (Fig. 3a–d). Immunohistochemical staining images showed the discontinuous part of the kidney capsule, indicating the resection area (white arrowheads in Fig. 3d). H&E and DAPI staining demonstrated that cells fully infiltrated the organ-derived matrix (Fig. 3a and c). Fibril formation in the matrix was examined using immunohistochemical staining of vimentin, a mesenchymal marker for fibrotic and regenerative wound repair, and α-smooth muscle actin (αSMA), a marker of myofibroblasts^23–27^. In the matrix, vimentin-positive renal tubular cells were observed, while the renal parenchyma and the residual kidney sutured to the matrix was negative for αSMA staining (Fig. 3c). Conversely, the PN kidney was negative for vimentin strongly positive for αSMA (Fig. 3d). αSMA staining revealed that the fibrotic tissue was formed in the PN kidney, while the formation of the fibrotic tissue was inhibited in the matrix and the residual kidney^23^. The negative expression of vimentin in the fibrous PN kidney resulted from the necrosis of renal tubular cells, as observed by their weak DAPI staining (Fig. 3d). These histological images evidenced that the organ-derived extracellular matrix-induced renal wound repair unaccompanied by fibrillogenesis within both the matrix and the residual kidney.

To investigate the cause of non-fibrotic tissue formation in the matrix and the residual kidney, genes involved in wound repair were analyzed in each tissue (Fig. 3e, Supplementary Fig. 3, Supplementary Tables 3 and 4). Gene expression levels in the residual kidney did not differ significantly from those in the healthy kidney (red histograms in Fig. 3f). The expression level in the non-fibrotic matrix was distinct from that in the fibrotic PN kidney (blue histograms in Fig. 3f). The matrix presented the upregulated expression of ECM components, including collagen type I, III, and V, and that of ECM-remodeling enzymes (matrix metalloproteinases (MMP), such as collagenase). In addition, the expression of genes encoding inflammatory (interleukin 1: IL1) and anti-inflammatory (IL4, IL10) cytokines, and that of genes encoding fibroblast growth factor (FGF) and transforming growth factor-beta (TGFβ), which are related to regenerative wound repair, fibrosis, and epithelial-to-mesenchymal transition (EMT), were upregulated^24^.

### Regeneration of glomeruli, vessels, and renal tubules within the organ-derived matrix

**Fig. 4:**
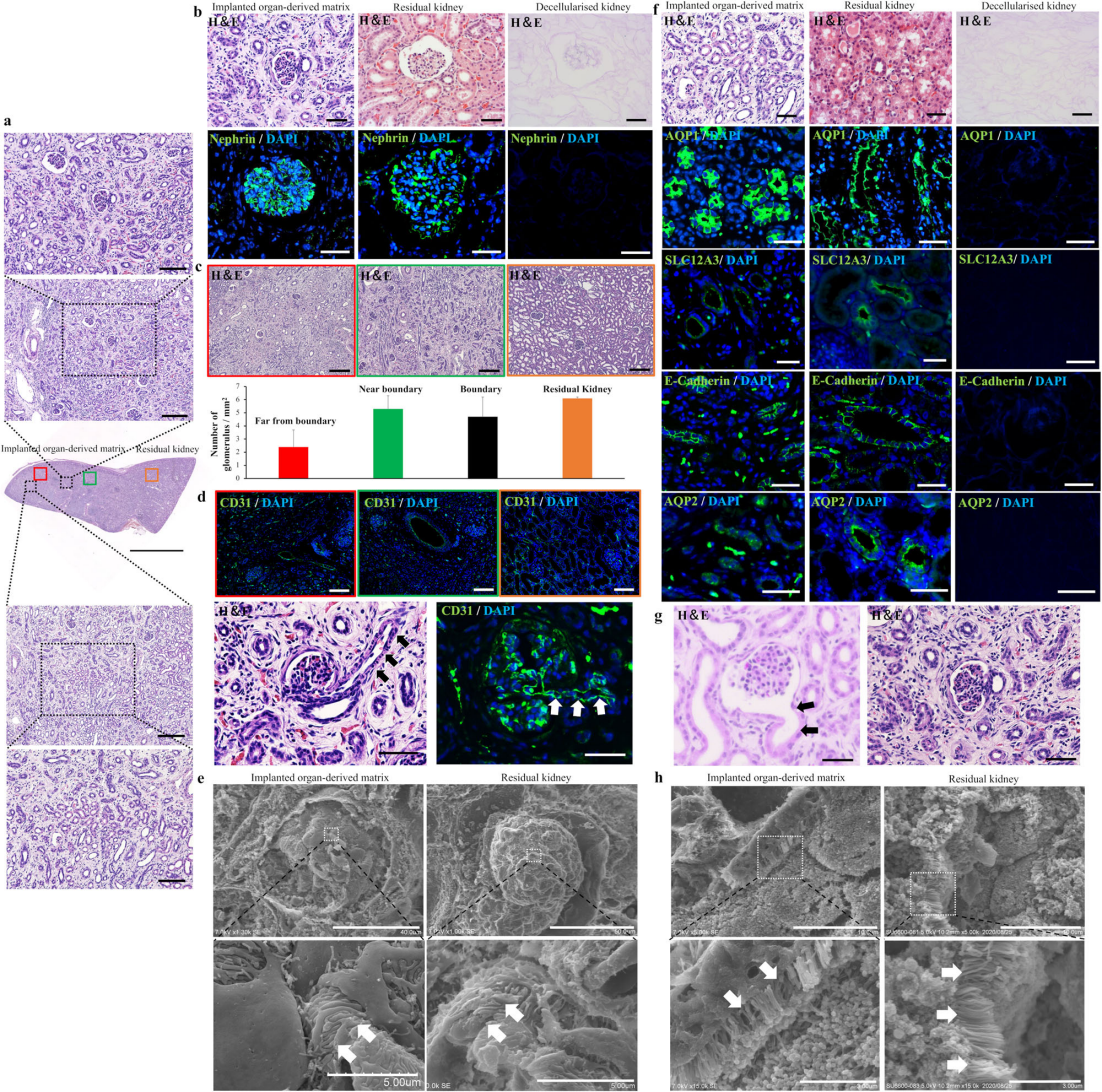
Regeneration of glomeruli, vessels, and renal tubules induced by the organ-derived matrix. **a** (middle) H&E staining montage image in the matrix sutured to the residual kidney. Red, green, and orange squares mark the region far from the boundary, near the boundary, and the residual kidney, respectively. Scale bar: 5 mm. (top and bottom) Enlarged images of the regions showing (top) glomerulus-like and (bottom) tubule-like structures. Scale bar: 200 and 100 μm for both images. **b** H&E images showing glomerulus-like structures in the matrix, matrix-sutured residual kidney, and decellularized kidney, and immunohistochemical images of nephrin staining in the kidneys counterstained with DAPI. Scale bar: 30 μm. **c** H&E staining showing a glomerulus structure and a histogram presenting the position-dependent number. Scale bar: 200 μm. The colors in the images and the histogram correspond to the areas represented by the colors in Fig. 4a. The black histogram corresponds to the area surrounded by the black square in Fig. 3a. Bars represent the mean ± SD, (*n* = 3). **d** (top) Immunohistochemical images of CD31 staining in the matrix counterstained with DAPI. The colors correspond to the areas represented by the colors in Fig. 4a. Scale bar: 100 μm. (bottom) H&E staining in the regions showing vessel-attached glomerulus structures in the matrix and CD31 staining counterstained with DAPI. The arrows show afferent arterioles. Scale bar: 50 μm. **e** SEM images showing glomerulus structures in the matrix and residual kidney. The arrows show foot processes. Scale bar: (top, left) 40 μm, (top, right) 50 μm, (bottom) 5 μm. **f** Magnified H&E staining images showing renal tubule-like structures in the matrix, residual kidney, and decellularized kidney, and immunohistochemical images of AQP1, SLC12A3, E-cadherin, and AQP 2 staining in the kidneys counterstained with DAPI. Scale bar: 30 μm. **g** H&E staining showing a juxtaglomerular apparatus-like structure in the matrix. The arrows indicate renal tubules. Scale bar: 50 μm. **h** SEM images of renal tubule structures in the matrix and residual kidney. The arrows indicate brush borders. Scale bar: (top) 10 μm, (bottom) 3 μm.

As shown in Fig. 3, the organ-derived matrix-induced wound repair unaccompanied by fibrillogenesis in the matrix. We further investigated whether glomeruli, vessels, or renal tubules, which are fundamental nephron-forming structures, were regenerated in the non-fibrotic matrix. Further analyses of H&E staining for the matrix and the residual kidney sutured to the matrix showed that the glomerulus-like structures with Bowman’s capsule-like components and infiltrated red blood corpuscles were formed over the matrix (Fig. 4a (top), 4b (top), Supplementary Fig. 4). The morphologies of the glomerulus-like structures were similar to those in the residual kidney (Fig. 4b (top)). Before the implantation of the organ-derived matrix, no cellular components were contained (Fig. 4b (top)). From immunohistochemical analyses, the observed glomerulus-like structures were markedly positive to the expression of nephrin, which is a major marker for podocytes, a type of glomerular cell (Fig. 4b (bottom))^28^. In addition, the expression pattern of nephrin was similar to that in the residual kidney, while nephrin was not expressed in the decellularized kidney (Fig. 4b (bottom)). These immunohistochemical analyses proved the regeneration of glomeruli in the matrix. The number of glomeruli within the matrix near the boundary was similar to that within the residual kidney and in the boundary region and was larger compared with the number in more distant areas from the boundary (Fig. 4c). The microstructures of the glomeruli formed in the matrix were also evaluated using SEM and transmission electron microscopy (TEM). The foot processes, characteristic of podocytes, were observed in the glomeruli within the matrix (white arrows in Fig. 4e (left))^29^. Furthermore, the ultrastructural evaluation using TEM allowed the characterization of podocytes, glomerular capillaries, and glomerular mesangial cells in the glomeruli within the matrix, as observed in the residual kidney (Supplementary Fig. 5)^29^. In addition to the high expression of nephrin, the vascular endothelial cell marker, CD31, was highly expressed in the matrix and the residual kidney (Fig. 4d (top)), while the expression of CD31 was negative around the resection area in the PN kidney (Supplementary Fig. 6). The regenerated vascular structure and afferent arteriole-like structure was also observed inside glomeruli in the matrix through H&E and CD31 staining, indicating the regeneration of continuous structures between glomeruli and vessels in the matrix (Fig. 4d (bottom)). It was expected that blood flow was supplied into the matrix through the regenerated vascular network structure.

From H&E staining, renal tubule-like structures were observed over the matrix (Fig. 4a (bottom), 4f). The structures comprised cuboidal cells and were similar to those in the residual kidney (Fig. 4f). To examine the regeneration of renal tubules in the matrix, we immunohistochemically analyzed the formation of proximal and distal renal tubules and renal collecting ducts using each specific cellular marker, namely AQP1 for proximal tubules, SLC12A3 and E-cadherin for distal tubules, and AQP2 for collecting ducts. AQP1-, SLC12A3-, E-cadherin-, and AQP2-positive cells were observed in the matrix (Fig. 4f). The expression patterns of the markers were similar to those in the residual kidney, although the luminal structures composed of positive cells were smaller in size compared with those in the residual kidney. The decellularized kidney as negative control did not express the cellular markers (Fig. 4f). The brush borders, characteristic of proximal renal tubules, were observed in the matrix using SEM, although they were shorter than those in the residual kidney (Fig. 4h, Supplementary Fig. 7). In addition, it was likely that the assembly composed of proximal tubular cells took an amorphous structure in the matrix (Fig. 4h). Immunohistochemical and SEM analyses exhibited that renal tubules regenerated inside the matrix, but the structures did not relatively mature to those in the residual kidney. Furthermore, the formation of juxtaglomerular apparatus-like structures was observed using H&E staining (Fig. 4g), suggesting the regeneration of continuous structures comprising glomeruli, vessels, and renal tubules in the matrix.

### Expression of renal progenitor markers in the organ-derived matrix

**Fig. 5:**
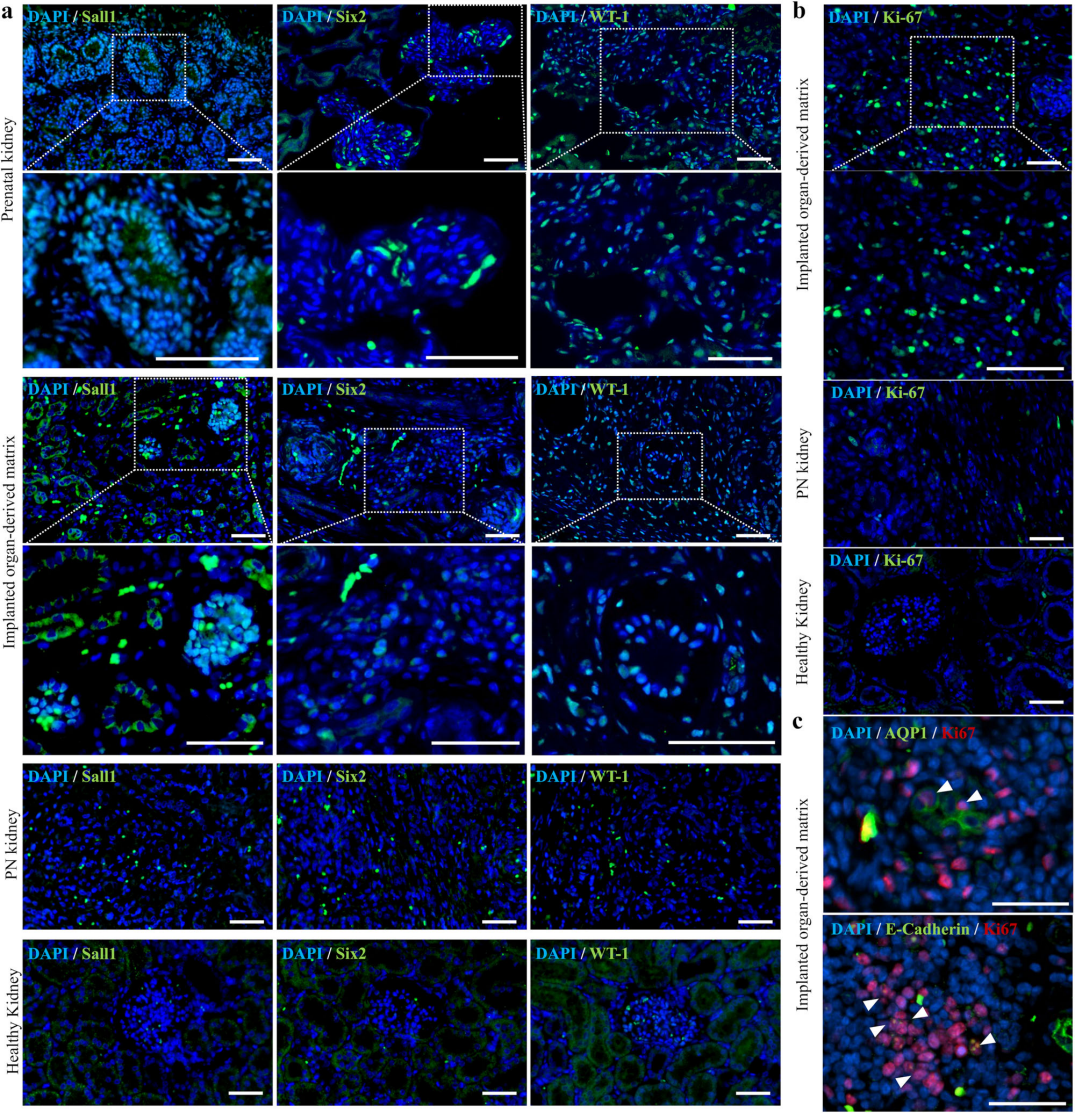
Expression of renal progenitor markers and proliferating cell markers in the organ-derived matrix. **a** Sall1, Six2, WT-1, and DAPI staining in the prenatal kidney, the implanted organ-derived matrix, the PN kidney 28 days postsurgery (control), and the healthy kidney. Scale bar: 100 μm. **b** Ki67 and DAPI staining in the implanted organ-derived matrix, the PN kidney 28 days postsurgery (control), and the healthy kidney. Scale bar: 100 μm. **c** (top) AQP1 and (bottom) E-cadherin staining of DAPI- and Ki67-stained cells in the matrix. Scale bar: 50 μm. The white arrowheads show the AQP1- or E-cadherin-positive cells.

The implanted organ-derived matrix functioned as an origin for the regeneration of characteristic nephron structures, including glomeruli, vessels, renal tubules, and their complexes. In general, as factors driving such regenerative wound repair, the cellular activities, such as the infiltration of stem/progenitor cells and the expression of unique immune systems in organ-derived matrices, have been studied^11,21,22^. Recent studies have reported that renal progenitor cells were isolated from endogenous mammalian kidneys^30,31^. Renal progenitor cells within organ-derived matrices may be a dominant factor for renal regeneration. Thus, the expression levels of typical renal progenitor markers, including Sall1 and Six2 for nephrons and WT-1 for podocytes, were immunohistochemically analyzed in the experimental groups as well as the prenatal kidney. As a positive control, the three types of progenitor markers were markedly expressed in the prenatal kidney. These were also expressed in the organ-derived matrix (Fig. 5a, Supplementary Fig. 8), while no expression was confirmed in the PN and healthy kidneys. Furthermore, the cells forming the luminal structure inside the matrix were positive for WT-1 (Supplementary Fig. 9). In addition to renal progenitor markers, the expression of a proliferating cell marker, Ki67, was examined. More Ki67-positive cells were detected in the matrix compared with the PN and healthy kidneys (Fig. 5b). Some Ki67-positive cells co-expressed AQP1 or E-cadherin, the respective markers for proximal and distal renal tubules, indicating that renal tubule cells in the matrix maintained their proliferation capability (Fig. 5c). These immunohistochemical staining results showed that the matrix was rich in renal progenitor and proliferative cells in contrast to the PN and healthy kidneys.

### Evaluation of blood flow inside the organ-derived matrix

**Fig. 6:**
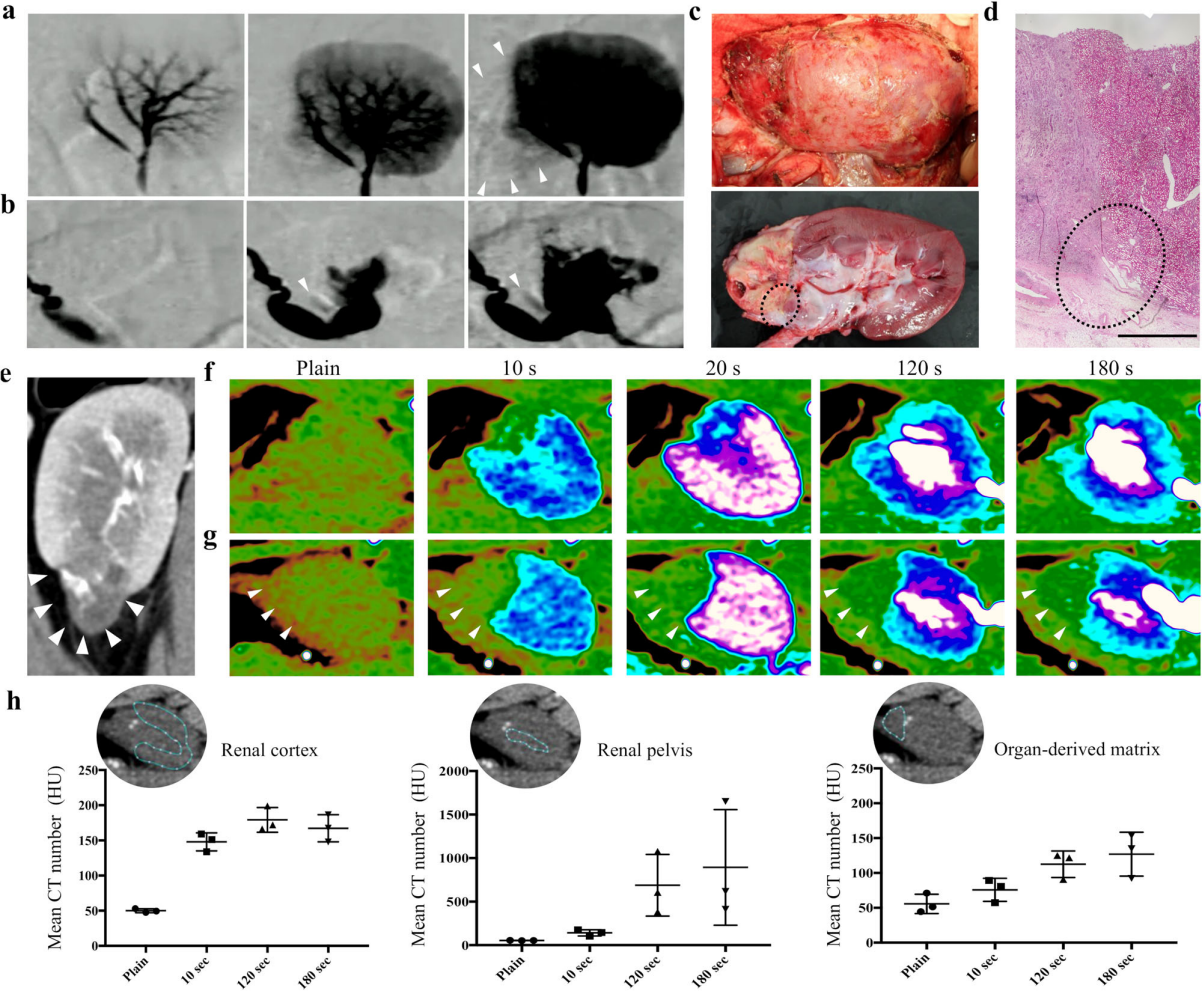
CT, angiography, and urography of the organ-derived matrix. **a**, **b** Time lapse of the **a** angiography and **b** urography images of the matrix-implanted kidney. The white arrowheads show the regions where the contrast was enhanced in the matrix. **c** Photographs of (top) a kidney containing an organ-derived matrix sutured to residual kidney before harvest 28 days post-implantation and (bottom) the harvested and sagittally resected kidney. The direction of the kidneys is identical to that of the kidneys imaged in Fig. 6a and b. **d** H&E staining of the area surrounded by the black-dotted circle in Fig. 6c. The area within the black-dotted circle in Fig. 6d shows the region where the matrix, residual kidney, and fused renal capsule are closely packed. Scale bar: 2 mm. **e** CT images of the matrix-implanted kidney. The white arrows show the matrix. **f**, **g** Time course of the CT scan images for **f** the PN kidney and **g** the matrix-implanted kidney. The white arrowheads show the matrix. **h** Time course of the mean CT number in the renal cortex, renal pelvis, and matrix in the implanted kidney. The CT number in ROI was estimated using OsiriX Lite. The white-dotted blue circles indicate ROI in the renal cortex, renal pelvis, and matrix. Bars represent the mean ± SD, (*n* = 3).

To examine the supply of blood flow into the matrix through the regenerated vessels, contrast-enhanced angiography and computed tomography (CT) were performed 28 days after implantation. Angiography showed a slightly enhanced contrast in a part of the matrix (white arrowheads in Fig. 6a), which indicated active blood flow into the matrix. This was supported by the slightly reddish matrix confirmed via anatomical evaluation (Fig. 6c) and the formation of blood vessels observed in the histological image around the suture area (Fig. 6d). The impaired blood flow around the kidney-resected areas was imaged using dynamic CT (Fig. 6f). In contrast, a gradual enhancement of image contrast and CT number was observed in the matrix-implanted area from 10 s after the intravenous administration of a contrast agent, although the CT number drastically increased in the renal cortex 10 s after the administration, followed by its increase in the renal pelvis (Fig. 6e, g, h). The increase in the CT number in the matrix confirmed the supply of blood flow into the matrix (Fig. 6h). An influx of a contrast agent inside the matrix was also observed using urography. As shown in Fig. 6b, the contrast was weakly enhanced around the matrix. Because the matrix-implanted kidney formed the closely packed structure composed of the matrix, residual kidney, and fused renal capsule, as observed in the histological staining (black-dotted circle in Fig. 6d), the enhancement of contrast in these images was not due to the leakage of the contrast agent into the abdominal cavity.

## Discussion

Renal regeneration has not been reported under physiological conditions in adult mammals. In the present study, we demonstrated that an organ-derived extracellular matrix incorporating an array of signaling molecules in situ induced porcine kidney regeneration. To induce the regeneration, a porcine kidney was partially resected, then the organ-derived matrix was sutured onto the resected kidney. Twenty-eight days after implantation, nephron regeneration was observed in the matrix based on wound repair unaccompanied by fibrillogenesis. Immunohistochemical analyses showed the emergence of renal progenitor cells in the matrix, indicating that the matrix served as a cellular niche for kidney regeneration. Furthermore, active blood flow was supplied inside the matrix. The matrix, with suitable ECM components and signaling molecules, provided a structural and functional foundation for in situ kidney regeneration.

The kidney-derived extracellular matrix prepared in this study did not possess any cellular component, as demonstrated via histological analyses. H&E-, Azan-, and DAPI-stained histological images, as well as SEM images, showed the absence of cytoplasmic and nuclear components in the matrix, which has been typically observed in decellularized organ scaffolds^12,18,22,32,33^. Recent advances in acellular bio-scaffolds and recellularization techniques provide a regenerative potential to complex organs, such as kidney and liver, in small mammals^7,12^. Previously, we reported that the extracellular matrices derived from the porcine heart, pancreas, and liver provide potential applications for organ regeneration^15,18,32^. The potential of a porcine kidney-derived matrix for kidney regeneration has been reported in a mouse model^33^. The main component of acellular bio-scaffolds is an organ-derived ECM, which provides structural support and regulates niche signaling during organ regeneration and neogenesis^34^. Laminin and collagen type IV are crucial ECM components of glomerular basement membranes. Laminin is necessary for glomerulogenesis and glomerular vascularization^35^. Null mutations in laminin β2 result in congenital nephrotic syndrome^36,37^. Collagen type IV mutations cause hereditary glomerulonephritis and ESRD^38^. Our SDS-based decellularization protocol maintained the functional renal ECM components, such as collagen type IV and laminin. The renal ECM proteins in the organ-derived matrix should take advantage of the development and maturation of the glomerular structure.

After implantation of the organ-derived matrix onto the surface of a PN-treated kidney, it was observed from a series of immunohistochemical analyses that fibrotic tissues were not formed inside the matrix and the adjoining residual kidney. In contrast, robust fibrotic tissues were formed over the PN kidney. These analyses provided evidence that the matrix-induced renal wound repair unaccompanied by fibrillogenesis in both the matrix and residual kidney. Previous studies have reported that vimentin expression is needed for the development of EMT-associated renal fibrosis^25,27^. In contrast, it was also suggested that vimentin is a marker of regenerative activity in renal tubules^26^. The expression of vimentin in the matrix may lead to regenerative wound repair in the matrix due to transient EMT activation^24^. It has also been considered that the differences in the induction of injury-triggered regenerative wound repair or pathologic fibrosis are regulated by signaling components and enzymes, including FGF, epidermal growth factor (EGF), TGFβ, hepatocyte growth factor (HGF), and MMP, which are commonly related to EMT^24,39^. The upregulated expression levels of genes encoding FGF and TGFβ in the non-fibrotic matrix led to cellular proliferation and re-epithelialization needed for regenerative wound repair, as observed by the expression of Ki-67 in the matrix^24^. In addition, the expression levels of genes associated with ECM structural components, including collagen type I, III, and V, were upregulated in the matrix, although their collagen types are renal fibrosis-related interstitial ones^40,41^. The upregulation of both ECM- and MMP-associated genes may demonstrate MMP-induced remodeling of ECM components in the matrix, not their degradation for fibrillogenesis. A recent study has reported that a bio-scaffold-induced type II immune response, characterized by IL4, promotes non-fibrotic wound repair, supporting the upregulated IL4 expression in the matrix^22^.

The further histological and immunohistochemical analyses revealed that glomeruli, renal tubules, and vessels, characteristic of nephrons, were regenerated in the nonfibrotic matrix. Barrier and filtration functions of glomeruli are highly regulated by podocytes, glomerular endothelium, and glomerular basement membranes^42^. Podocytes have an interdigitating network (foot process) and are interconnected by the network (slit diaphragm)^43^. Foot processes play an essential role in glomerular barrier functions and regulate protein loss and blood filtration. The formation of foot processes in the matrix was observed from SEM images, which provides the potential of size-selective barrier and filtration functions for the matrix. The regeneration of renal tubules in the matrix was ensured based on the characterization of proximal and distal renal tubules and renal collecting ducts with each specific cellular marker^44,45^. The renal tubule cells expressing the markers were abundant within the matrix and the formation of continuous lumen structures was confirmed. In addition, brush borders with an abundance of microvilli were regenerated on the surfaces of the tubule structures, although they were immature compared to the residual kidney. Microvilli cover the apical membranes of proximal tubules and enhance water resorption^46^. The regenerated renal tubules potentially resorb water. The histological and immunohistochemical analyses also demonstrated the regeneration of vascular structure in the matrix. Notably, an afferent arteriole-like structure connected with regenerated glomeruli and a juxtaglomerular apparatus-like structure were observed, indicating the regeneration of continuous nephron structures.

Angiography and dynamic CT allowed imaging active blood flow in a part of the implanted matrix. The gradual increase in the CT number in the matrix might be due to the low circulation rate of the contrast agent. Histological analysis did not show evidence of remarkable blood congestion within the matrix. The regeneration of glomeruli observed within the matrix may induce their filtration functions, leading to reduced excretion of the contrast agent.

A part of the cells inside the matrix expressed renal progenitor markers, including Sall1, Six2, and WT-1. A previous study showed that renal progenitor cells are non-viable in the adult kidney because of the lack of their niche^47^. In our porcine model, the matrix served as a niche for renal progenitor cells and induced their engraftment. However, the origin of the progenitor cells is not clear at present. Histological analyses showed that the number of regenerated glomeruli decreased in more distant regions from the boundary between the matrix and the residual kidney. Thus, the matrix may stimulate the emergence of renal progenitor cells in the residual kidney, followed by their infiltration into the matrix.

Our porcine model demonstrated that the implanted organ-derived extracellular matrix induced the structural regeneration of glomeruli, renal tubules, and vessels, and supported the regeneration of their continuous structures as well as the resultant active blood flow. The regenerated glomeruli and renal tubules involved interdigitating foot processes and brush borders with microvilli, respectively. In addition, the progenitor and proliferating cells were abundant inside the matrix. These experimental results provided evidence that the matrix served as a cellular niche for nephron regeneration and the regenerated nephron structures originated from the matrix. A limitation of the present study is the lack of investigation on the mechanism of the nephron regeneration in the matrix. Although various medical applications potentially place organ-derived extracellular matrices in proximity to damaged organs or tissues, little is known concerning the mechanism of regenerative wound repair induced in the matrices. Interestingly, fibrotic tissue was also not formed in the residual kidney sutured to the matrix. The matrix may stimulate the adjoining kidney, leading to the recruitment of the progenitor and proliferating cells into the matrix. Further research is warranted to elucidate the origin of the progenitor and proliferating cells observed in the matrix and the influence of the matrix on surrounding damaged organs. The present study provided preclinical evidence that the organ-derived extracellular matrix, which is composed of ECM components and signaling molecules, can induce in situ renal regeneration. Such matrices prepared without any external cell seeding can potentially help avoid rejection by the immune system, leading to the induction of structural and functional regeneration of damaged organs due to their matrix-assisted self-repair and spontaneous healing abilities. The realization of the organ regeneration presented in this study largely contributes not only to the establishment of a platform for renal regeneration and therapy but also to the advancement for the development of various organs-derived extracellular matrices as scaffolds for organ regeneration.

## Methods

### Animals

Experimental procedures and protocols were approved by the Animal Ethics Committee of Keio University Tokyo, Japan, (approval number: 15015) and were conducted in accordance with the Guide for the Care and Use of Laboratory Animals (National Institutes of Health, Bethesda, MD, USA). Female domestic pigs weighing approximately 20 kg and prenatal pigs were purchased from Sankyo Labo Service Corp., Inc., Tokyo, Japan.

### Harvest of a porcine kidney and its decellularization

Porcine kidneys were harvested under general anesthesia. Briefly, pigs were sedated using an intramuscular injection cocktail comprising 0.02 mg kg^–1^ medetomidine and 1 mg kg^–1^ midazolam. After confirmation of sedation, pigs were intubated with a tracheal tube (outer diameter: 6.5 mm) and the anesthesia was maintained via 2% isoflurane inhalation with an endotracheal tube. Buprenorphine suppository (0.2 mg head^–1^) was rectally inserted for preoperative analgesia. The operating field was disinfected with 10% w/v povidone. A sterile drape was aseptically affixed to the abdomen. All surgeries were conducted under sterile conditions. Median incisions were performed with a scalpel, and the right and left renal arteries of the pigs were isolated and cannulated with 18 G indwelling needles. The renal arteries, renal veins, and ureters were cut to harvest the kidneys, and the pigs were euthanized by exsanguination. The harvested kidneys were either treated as healthy kidneys (sample 2 in Fig. 2g) or were stored over 24 h at −80 °C for decellularization. Before decellularization, the kidneys were stored at 4 °C for 18 h, then maintained at room temperature for 1 h. The kidneys were then perfused with phosphate-buffered saline (PBS; FUJIFILM Wako Pure Chemical Corp. Osaka, Japan) for 2 h (10 mL min^–1^) through the cannulated renal artery using a tubing pump (Masterflex L/S with Easy-Load Pump Head III; Yamato Scientific Co. Ltd., Tokyo, Japan) and tubes (Masterflex Tube L/S16; Yamato Scientific Co. Ltd.). Thereafter, samples were perfused with 0.5% SDS (FUJIFILM Wako Pure Chemical Corp.) for 4 h (10–20 mL min^–1^) for decellularization. Acellular kidneys were perfused with PBS for 3 h (20 mL min^–1^) for washing. They were sterilized using 25 kGy γ-irradiation (RADIA Industry Co. Ltd., Gunma, Japan), then were stored at 4 °C before use.

### Protein analysis of the organ-derived extracellular matrix

The decellularized tissue soaked in PBS was subdivided with a scalpel. Ten milligrams of the subdivided tissue were digested in an aqueous solution (0.001% Trypsin, 10 mM Tris-HCl (pH: 8.8), 0.005% n-octyl glucopyranoside, 0.7 M guanidine hydrochloride) for 12 h. The digested peptides from the tissues were reduced using 5 mM tris(2-carboxyethyl) phosphine (TCEP) at 65 °C for 30 min, then alkylated using 10 mM iodoacetamide at 25 °C for 30 min, followed by purification using a column (MonoSpin C18; GL Science K.K., Tokyo, Japan). Purified peptides (500 ng) were analyzed using a liquid chromatogram (Easy-nLC 1200; Thermo Fisher Scientific K.K., Tokyo, Japan) coupled with a high-resolution quadrupole-orbitrap mass spectrometer (Q-Exactive HF-X; Thermo Fisher Scientific K.K.). The peptides were separated using a C18-separation column (3 μm, 100 µm inner diameter ×12 cm; Nikkyo Technos, Co. Ltd., Tokyo, Japan), where the mobile phases A and B consisted of 0.1% formic acid in water and 0.1% formic in 80% acetonitrile, respectively. The peptide solutions flowed at a constant rate of 300 nL min^–1^ in mobile phase B at a gradient of 5–40%. The eluent was directly introduced into the liquid chromatography-mass spectroscopy (LC-MS) instrument. MS spectra were recorded in a range of 380 to 1500 m/z. The resolution for MS/MS was set to 15,000 at 200 m/z. All data were analyzed using the Proteome Discoverer 2.2 (Thermo Fisher Scientific K.K.). All MS/MS spectra were searched based on protein sequences presented on the *Sus scrofa* protein database (NCBI: txid9823). The false discovery rate was set at 1% for the peptide-spectrum match. Peptide level quantitation was performed and label-free quantification was based on the signal intensity of precursors. Among the identified proteins, the proteins related to “extracellular” and “membrane” determined via Gene Ontology were extracted and are summarized in Supplementary Tables 1 and 2, where 100 proteins are shown in descending order of their detection.

### Control and organ-derived matrix-sutured model

The pigs were allowed to fast for 12 h until surgery with *ad libitum* access to water. Surgeries were performed aseptically under general anesthesia according to a previously published method^19^. Midline incisions were performed, then the right renal arteries and veins were isolated. About one-third of each caudal kidney was resected, and bleeding was controlled with an electric device, such as a bipolar system. The open collecting systems were closed with bladed absorbable sutures (4-0 vicryl; Johnson & Johnson K.K., Tokyo, Japan). The organ-derived matrix was trimmed to a similar size as the resected kidney and was sutured onto the resected surface of the residual kidney with bladed absorbable sutures (4-0 vicryl) at approximately 1.5 cm intervals (Fig. 2a). As a control, the kidney treated with a similar resection and bleeding method was used without the suture of an organ-derived matrix (Fig. 2b). After abdominal closure, the pigs were awakened from anesthesia and were administered with an analgesic (0.2 mg buprenorphine head^–1^ day^–1^) and an antibiotic (20 mg kg^–1^ cefalexin (Keflex), Shionogi & Co. Ltd., Osaka, Japan) for 1 week. After 28 days, the pigs were sacrificed under general anesthesia, and their kidneys (samples 1, 3, and 4 in Fig. 2g) were harvested. The resection rate was calculated as follows: resection rate (%) = major axis of the resected kidney (mm) × 100 (%) / major axis of the native kidney (mm). After harvesting the kidneys, the major axes of both the control and matrix-sutured kidneys were also measured.

### Histological analysis

After the harvested kidney samples were fixed with 4% paraformaldehyde (PFA) for 24 h, they were embedded in paraffin. They were then sliced into 4, 2, and 3 μm thick sections using a standard tissue processor. The first slice was stained with H&E (Sigma-Aldrich Co. LLC., MO, USA). The second was stained with Azan (Muto Pure Chemicals Co. Ltd., Tokyo, Japan). The third was used in immunohistochemical analysis. H&E and Azan staining were performed using standard protocols. Macro-scale montage images of H&E staining were created by merging low-magnification field images using Adobe Photoshop (Adobe Inc., CA, USA) and BZ-X800 viewer (KEYENCE Corp., Osaka, Japan).

### Immunohistochemical analysis

The sample sections were deparaffinized and rehydrated with xylene, diluted ethanol aqueous solutions (100, 90, 80, and 70%), and distilled water. The antigens in each sample were retrieved with citrate buffer (Agilent Technologies, CA, USA) at 120 °C for 15 min. The samples were stored at room temperature for 30 min. Thereafter, samples were incubated with blocking solution (N102; NOF Corp., Tokyo, Japan) for 30 min at room temperature to inhibit nonspecific reactions. The blocked samples were incubated with a primary antibody solution at 4 °C overnight. Primary antibodies used were CD31 (50:1; Abcam, Cambridge, UK), nephrin (1,000:1; Abcam), AQP1 (500:1; Abcam), E-cadherin (50:1; Abcam), fibronectin (1,000:1; Abcam), Ki67 (50:1; BD Biosciences, NJ, USA), collagen type IV (500:1; Abcam), laminin (50:1; Abcam), WT-1 (10:1, Aviva Systems Biology, CA, USA), Sall1 (50:1, Biorbyt, Cambridge, UK), Six2 (200:1, Proteintech Group, Inc., IL, USA), collagen type I (50:1, Abcam), vimentin (100:1, Dako, CA, USA), αSMA (200:1, Sigma-Aldrich Co. LLC.), SLC12A3 (50:1, Bioss Antibodies, MA, USA), and AQP2 (50:1, Bioss Antibodies). After washing, secondary antibodies (DyLight 488, Alexa 488, or Alexa 568; 1,000:1, Abcam) were added and incubated for 1 h at room temperature. The slides were washed and mounted with a 4’,6-diamidino-2-phenylindole (DAPI) solution (Vector Laboratories, Inc., CA, USA). Images were acquired using a fluorescence microscope (EVOS Cell Imaging System; Thermo Fisher Scientific K.K., BZ-X810; KEYENCE Corp., ECLIPSE Ni, and DS-Ri2; Nikon Corp., Tokyo, Japan).

### SEM

The kidney was harvested 28 days after the implantation of the organ-derived matrix. In the kidney, the parts of the matrix and residual kidney were fixed with 2.5% glutaraldehyde in HEPES for 24 h, then rinsed with HEPES. After the samples were subjected to 1% tannic acid at 4 °C for 2 h, they were fixed with 1% OsO_4_ in HEPES for 2 h. The fixed samples were dehydrated with a series of diluted ethanol aqueous solutions (50–100%), then substituted with *t*-butyl alcohol. After freeze-drying, they were osmium-coated. SEM images were obtained using a field emission-scanning electron microscope (SU6600; Hitachi High-Technologies Corp., Tokyo, Japan).

### TEM

Similar to SEM experiments, samples were harvested. The samples were fixed with 2.5% glutaraldehyde in HEPES for 24 h. After the samples were rinsed with HEPES, they were subjected to 1% tannic acid at 4 °C for 1–2 h. The samples were then fixed with 1% OsO_4_ in HEPES for 2 h. The fixed samples were dehydrated with a series of diluted ethanol aqueous solutions (50–100%). They were substituted overnight with epoxy resin (Okenshoji Co., Ltd., Tokyo, Japan) graded with *n*-butyl glycidyl ether. On the following day, the samples were embedded into 100% epoxy resin at 4 °C for 48 h, followed by polymerization at 60 °C for 72 h. Ultrathin sections at 70 nm thickness were prepared on copper grids (Veco Specimen Grids; Nisshin-EM, Tokyo, Japan) using an ultramicrotome (Leica UC7; Leica Biosystems, Wetziar, Germany) and stained with uranyl acetate and lead citrate each for 10 min. TEM images were obtained under 100 kV using JEM-1400Plus (JEOL Ltd., Tokyo, Japan).

### CT, angiography, and urography

Plain- and contrast-enhanced abdominal CT scans (Optima CT660 Advance; GE Healthcare Japan Corp., Tokyo, Japan) were performed 28 days after the implantation of the organ-derived matrix. Scanning was performed under general anesthesia using the following parameters: 120 kV, 150 mA, 40 mm beam collimation, 1 s rotation time, and 1.25 mm slice thickness. According to a phase-contrast injection protocol, a power injector (Stellant CT Injection System; Nihon Medrad K.K., Osaka, Japan) was used. Iopamidol (Iopamiron 300 injectable; Bayer Yakuhin Ltd., Osaka, Japan) was administered intravenously at 600 mg iodine per kg body weight for 15–20 s. Scanning was performed before (plain) and 10, 20, 120, and 180 s after administration of the contrast agent. All data were obtained in Digital Communications in Medicine (DICOM) format and were analyzed using OsiriX Lite ver. 10.0.2 (Pixmeo Sàrl, Bernex, Switzerland). Cross-sectional color images were obtained using the Color Look-Up Tables (CLUT) function with the French setting. Regions of interest (ROI) for the renal cortex, renal pelvis, and implanted organ-derived matrix were used to calculate the mean and standard deviation (SD) values of the CT numbers (Houncefield unit, HU). Each ROI was set manually.

Angiography and urography were performed using mobile C-arm equipment (ARCADIS Avantic; SIEMENS Healthcare K.K. Tokyo, Japan). An iodinated intravenous contrast agent (Omnipaque 350; Daiichi Sankyo Co. Ltd., Tokyo, Japan) was injected through the renal artery and ureter.

### Gene expression

After harvest, the kidney samples were immediately immersed in RNA*later* stabilization solution (Thermo Fisher Scientific K.K.) for gene expression analysis. Samples were stored at −20 °C until total RNA extraction. Each sample was homogenized, then total RNA was isolated with a commercially available column-based purification kit (RNeasy Mini; QIAGEN K.K., Tokyo, Japan). RNA was measured using NanoDrop spectrophotometer (Thermo Fisher Scientific K.K.). Complementary DNA (cDNA) was synthesized from 1 μg total RNA per sample with PrimeScript RT Master Mix (TaKaRa Bio Inc., Shiga, Japan) and QuantiTect Reverse Transcription Kit (QIAGEN K.K.). Gene expression was analyzed using a commercially available mRNA array kit (RT^2^ Profiler PCR array; QIAGEN K.K.) combined with RT^2^ SYBR Green qPCR Mastermix (QIAGEN K.K.) or PowerUP SYBR Green Mastermix (Thermo Fisher Scientific K.K.) using ViiA7 real-time PCR system (Thermo Fisher Scientific K.K.). The PCR array contained primers for genes encoding ECMs, cellular adhesion molecules, inflammatory cytokines, chemokines, growth factors, and signal transduction proteins. The PCR array data were analyzed using the QIAGEN Data Analysis Center website (https://www.qiagen.com/ca/shop/genes-and-pathways/data-analysis-center-overview-page/). A clustergram for the genes was established automatically on the website.

### Statistics

Data were processed using GraphPad PRISM v. 7.0d for Mac (GraphPad Software Inc., CA, USA). Differences between pairs of groups, including resection rate (*n* = 4) and size of the sacrificed kidney (*n* = 4), were analyzed using the Mann-Whitney unpaired *t*-test. The resection rate and size of the sacrificed kidneys were determined from distinct samples. Two-sided statistical tests were performed in all analyses and significance was set at *P* < 0.05.

### Reporting summary

Further information on research design is available in the Nature Research Reporting Summary linked to this article.

## Supplementary information

Supplementary information

REPORTING SUMMARY

## Data Availability

The authors declare that all data supporting the findings of this study are available within the paper and its supplementary information.

## References

[1] Bertram JF, Douglas-Denton RN, Diouf B, Hughson MD, Hoy WE (2011). Human nephron number: implications for health and disease. Pediatr. Nephrol..

[2] Keller G, Zimmer G, Mall G, Ritz E, Amann K (2003). Nephron number in patients with primary hypertension. N. Engl. J. Med..

[3] Saxen L, Sariola H (1987). Early organogenesis of the kidney. Pediatr. Nephrol..

[4] Diep CQ (2011). Identification of adult nephron progenitors capable of kidney regeneration in zebrafish. Nature.

[5] Jha V (2013). Chronic kidney disease: global dimension and perspectives. Lancet.

[6] Wolfe RA (1999). Comparison of mortality in all patients on dialysis, patients on dialysis awaiting transplantation, and recipients of a first cadaveric transplant. N. Engl. J. Med..

[7] Song JJ (2013). Regeneration and experimental orthotopic transplantation of a bioengineered kidney. Nat. Med..

[8] Barkan D, Green JE, Chambers AF (2010). Extracellular matrix: a gatekeeper in the transition from dormancy to metastatic growth. Eur. J. Cancer.

[9] Sellaro TL, Ravindra AK, Stolz DB, Badylak SF (2007). Maintenance of hepatic sinusoidal endothelial cell phenotype in vitro using organ-specific extracellular matrix scaffolds. Tissue Eng..

[10] Nelson CM, Bissell MJ (2006). Of extracellular matrix, scaffolds, and signaling: tissue architecture regulates development, homeostasis, and cancer. Annu. Rev. Cell Dev. Biol..

[11] Badylak SF, Freytes DO, Gilbert TW (2009). Extracellular matrix as a biological scaffold material: Structure and function. Acta Biomater..

[12] Uygun BE (2010). Organ reengineering through development of a transplantable recellularized liver graft using decellularized liver matrix. Nat. Med..

[13] Kadota Y (2014). Mesenchymal stem cells support hepatocyte function in engineered liver grafts. Organogenesis.

[14] Yagi H (2013). Human-scale whole-organ bioengineering for liver transplantation: a regenerative medicine approach. Cell Transplant..

[15] Katsuki Y (2016). Endocrine pancreas engineered using porcine islets and partial pancreatic scaffolds. Pancreatology.

[16] Bonandrini B (2014). Recellularization of well-preserved acellular kidney scaffold using embryonic stem cells. Tissue Eng. Part A.

[17] Ross EA (2009). Embryonic stem cells proliferate and differentiate when seeded into kidney scaffolds. J. Am. Soc. Nephrol..

[18] Shimoda H (2019). Decellularized liver scaffolds promote liver regeneration after partial hepatectomy. Sci. Rep..

[19] Ong AM (2003). Bipolar needle electrocautery for laparoscopic partial nephrectomy without renal vascular occlusion in a porcine model. Urology.

[20] Gurtner GC, Werner S, Barrandon Y, Longaker MT (2008). Wound repair and regeneration. Nature.

[21] Anderson JM, Rodriguez A, Chang DT (2008). Foreign body reaction to biomaterials. Semin. Immunol..

[22] Sadtler K (2016). Developing a pro-regenerative biomaterial scaffold microenvironment requires T helper 2 cells. Science.

[23] Duffield JS (2014). Cellular and molecular mechanisms in kidney fibrosis. J. Clin. Invest..

[24] Stone RC (2016). Epithelial-mesenchymal transition in tissue repair and fibrosis. Cell Tissue Res..

[25] Yamashita S, Maeshima A, Nojima Y (2005). Involvement of renal progenitor tubular cells in epitherial-to mesenchymal transition in fibrotic rat kidneys. J. Am. Soc. Nephrol..

[26] Kriz W, Kaissling B, Le Hir M (2011). Epithelial-mesenchymal transition (EMT) in kidney fibrosis: fact or fantazy?. J. Clin. Invest..

[27] Wang Z (2018). Vimentin expression is required for the development EMT-related renal fibrosis following unilateral ureteral obstruction in mice. Am. J. Physiol. Ren. Physiol..

[28] Ronconi E (2009). Regeneration of glomerular podocytes by human renal progenitors. J. Am. Soc. Nephrol..

[29] Ichimura K (2015). Three-dimensional architecture of podocytes revealed by block-face scanning electron microscopy. Sci. Rep..

[30] Romagnani P, Lasagni L, Remuzzi G (2013). Renal progenitors: an evolutionary conserved strategy for kidney regeneration. Nat. Rev. Nephrol..

[31] Bussolati B (2005). Isolation of renal progenitor cells from adult human kidney. Am. J. Pathol..

[32] Kitahara H (2016). Heterotopic transplantation of a decellularized and recellularized whole porcine heart. Interact. Cardiovasc. Thorac. Surg..

[33] Hussein KH (2018). Biocompatibility and hemocompatibility of efficiently decellularized whole porcine kidney for tissue engineering. J. Biomed. Mater. Res. A.

[34] Watt FM, Huck WT (2013). Role of the extracellular matrix in regulating stem cell fate. Nat. Rev. Mol. Cell Biol..

[35] Miner JH (2011). Organogenesis of the kidney glomerulus: focus on the glomerular basement membrane. Organogenesis.

[36] Chen YM, Kikkawa Y, Miner JH (2011). A missense LAMB2 mutation causes congenital nephrotic syndrome by impairing laminin secretion. J. Am. Soc. Nephrol..

[37] Suh JH, Jarad G, VanDeVoorde RG, Miner JH (2011). Forced expression of laminin beta1 in podocytes prevents nephrotic syndrome in mice lacking laminin beta2, a model for Pierson syndrome. Proc. Natl Acad. Sci. U. S. A..

[38] Kashtan CE (2000). Alport syndromes: phenotypic heterogeneity of progressive hereditary nephritis. Pediatr. Nephrol..

[39] Eming SA, Martin P, Tomic-Canic M (2014). Wound repair and regeneration: mechanisms, signaling, and translation. Sci. Transl. Med..

[40] Alexakis C, Maxwell P, Bou-Gharios G (2006). Organ-specific collagen expression: implications for renal disease. Nephron Exp. Nephrol..

[41] Genovese F, Manresa AA, Leeming DJ, Karsdal MA, Boor P (2014). The extracellular matrix in the kidney: a source of novel non-invasive biomarkers of kidney fibrosis?. Fibrogenes. Tissue Repair.

[42] Chitra PS (2015). Growth Hormone Induces Transforming Growth Factor-Beta-Induced Protein in Podocytes: Implications for Podocyte Depletion and Proteinuria. J. Cell. Biochem..

[43] Grahammer F, Schell C, Huber TB (2013). The podocyte slit diaphragm−from a thin grey line to a complex signalling hub. Nat. Rev. Nephrol..

[44] Nouwen EJ, Dauwe S, van der Biest I, De Broe ME (1993). Stage- and segment-specific expression of cell-adhesion molecules N-CAM, A-CAM, and L-CAM in the kidney. Kidney Int..

[45] Georgas K (2008). Use of dual section mRNA in situ hybridisation/immunohistochemistry to clarify gene expression patterns during the early stages of nephron development in the embryo and in the mature nephron of the adult mouse kidney. Histochem. Cell Biol..

[46] Venkatachalam MA, Bernard DB, Donohoe JF, Levinsky NG (1978). Ischemic damage and repair in the rat proximal tubule: differences among the S1, S2, and S3 segments. Kidney Int..

[47] Hartman HA, Lai HL, Patterson LT (2007). Cessation of renal morphogenesis in mice. Dev. Biol..

