# Supplementary material for: An organ-derived extracellular matrix triggers in situ kidney regeneration in a preclinical model
Source: NPJ Regen Med. 2022 Feb 28;7:18. doi: 10.1038/s41536-022-00213-y (PMC8885654; doi:10.1038/s41536-022-00213-y)
Supplement: Supplementary file 1 — Supplementary information [file 41536_2022_213_MOESM1_ESM.pdf]

Supplementary Information

An organ-derived extracellular matrix triggers *in situ* kidney regeneration in a preclinical model

Kazuki Tajima, Hiroshi Yagi*, Toshinori Morisaku, Kotaro Nishi, Hiroko Kushige, Hideaki Kojima, Hisanobu Higashi, Kohei Kuroda, Minoru Kitago, Shungo Adachi, Tohru Natsume, Kumiko Nishimura, Mototsugu Oya and Yuko Kitagawa

*Corresponding author. Email: h_yagi@a3.keio.jp

Supplementary Fig. 1 The cellular framework was preserved in the decellularized kidney.

Supplementary Fig. 2 The decellularized kidney contained no DAPI-positive cells.

Supplementary Fig. 3 Clustergram in Fig. 3e.

Supplementary Fig. 4 Formation of the glomerulus-like structures with Bowman's capsule-like components and infiltrated red blood corpuscles within the organ-derived matrix.

Supplementary Fig. 5 Formation of Pod, Cap, and GMC in the glomeruli within the matrix.

Supplementary Fig. 6 The expression of CD31 was negative around the resection area in the PN kidney.

Supplementary Fig. 7 The brush borders in the matrix were shorter than those in the residual kidney.

Supplementary Fig. 8 The progenitor markers are expressed in the regions away from the boundary of the matrix and residual kidney.

Supplementary Fig. 9 The cells forming the luminal structure inside the organ-derived matrix were positive for WT-1.

Supplementary Table. 1 Residual proteins in the organ-derived matrix: list 1 (No. 1–50)

Supplementary Table. 2 Residual proteins in the organ-derived matrix: list 2 (No. 51–100)

Supplementary Table. 3 Genes analyzed in the kidneys: list 1

Supplementary Table. 4 Genes analyzed in the kidneys: list 2

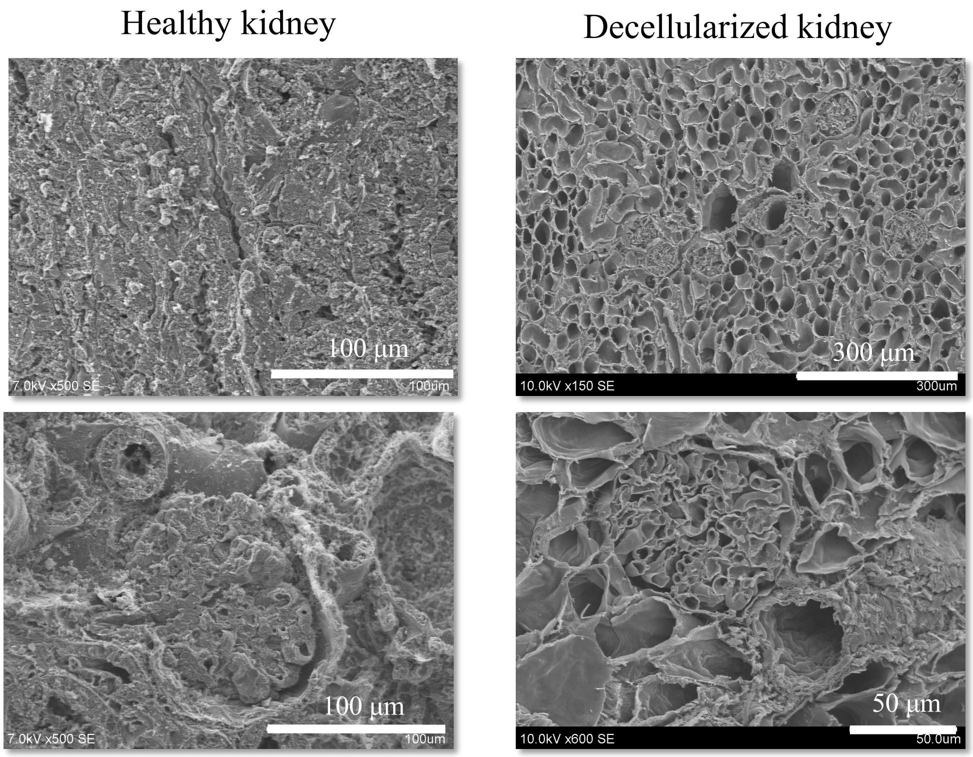

Supplementary Fig. 1 The cellular framework was preserved in the decellularized kidney.

SEM images of (left) a healthy kidney and (right) the decellularized kidney.

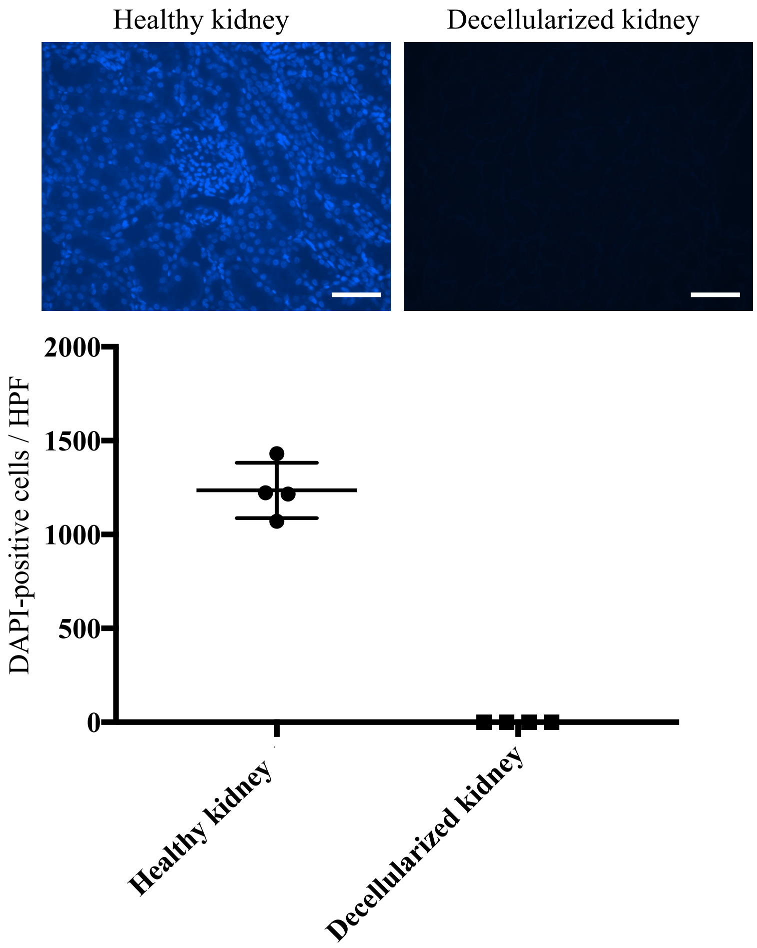

Supplementary Fig. 2 The decellularized kidney contained no DAPI-positive cells.

(top) Images of DAPI staining in a healthy and decellularized kidney and (bottom) the number of DAPI-positive cells in the kidney. Scale bar: 50 μm.

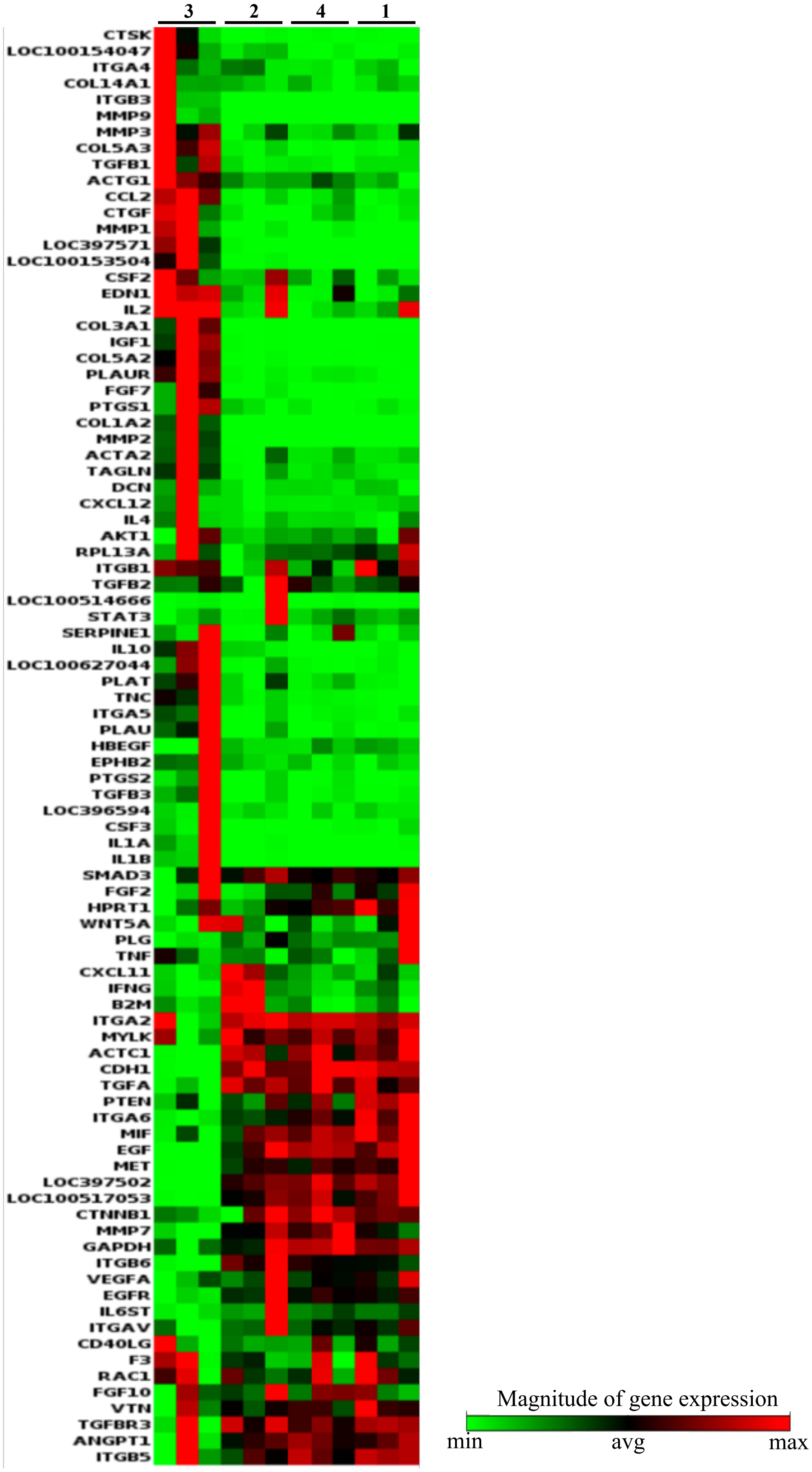

Supplementary Fig. 3 Clustergram in Fig. 3e.

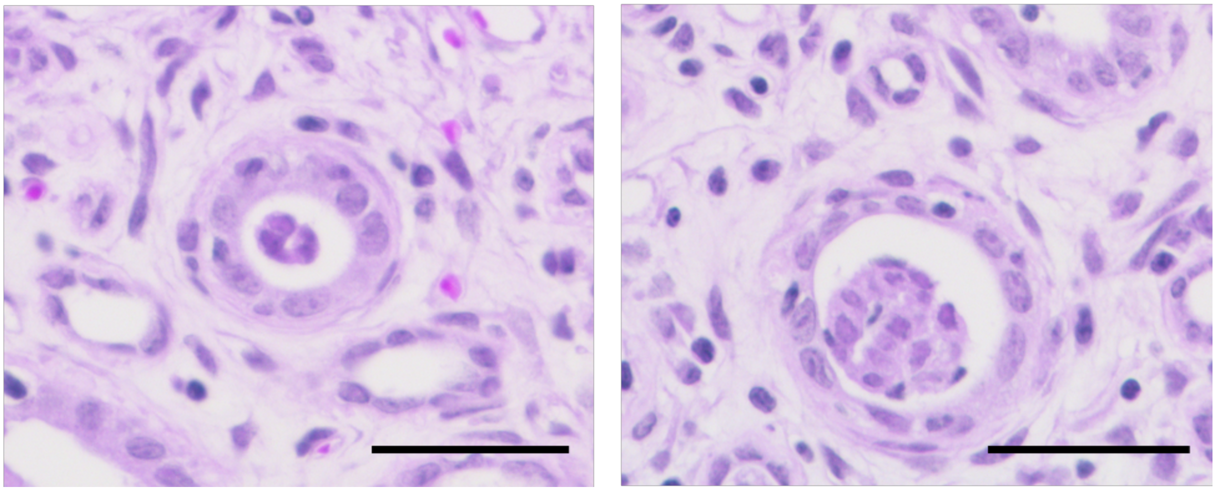

Supplementary Fig. 4 Formation of the glomerulus-like structures with Bowman's capsule-like components and infiltrated red blood corpuscles within the organ-derived matrix.

Typical H&E staining images of the glomerulus-like structures in the organ-derived matrix. Scale bar: 30 μm.

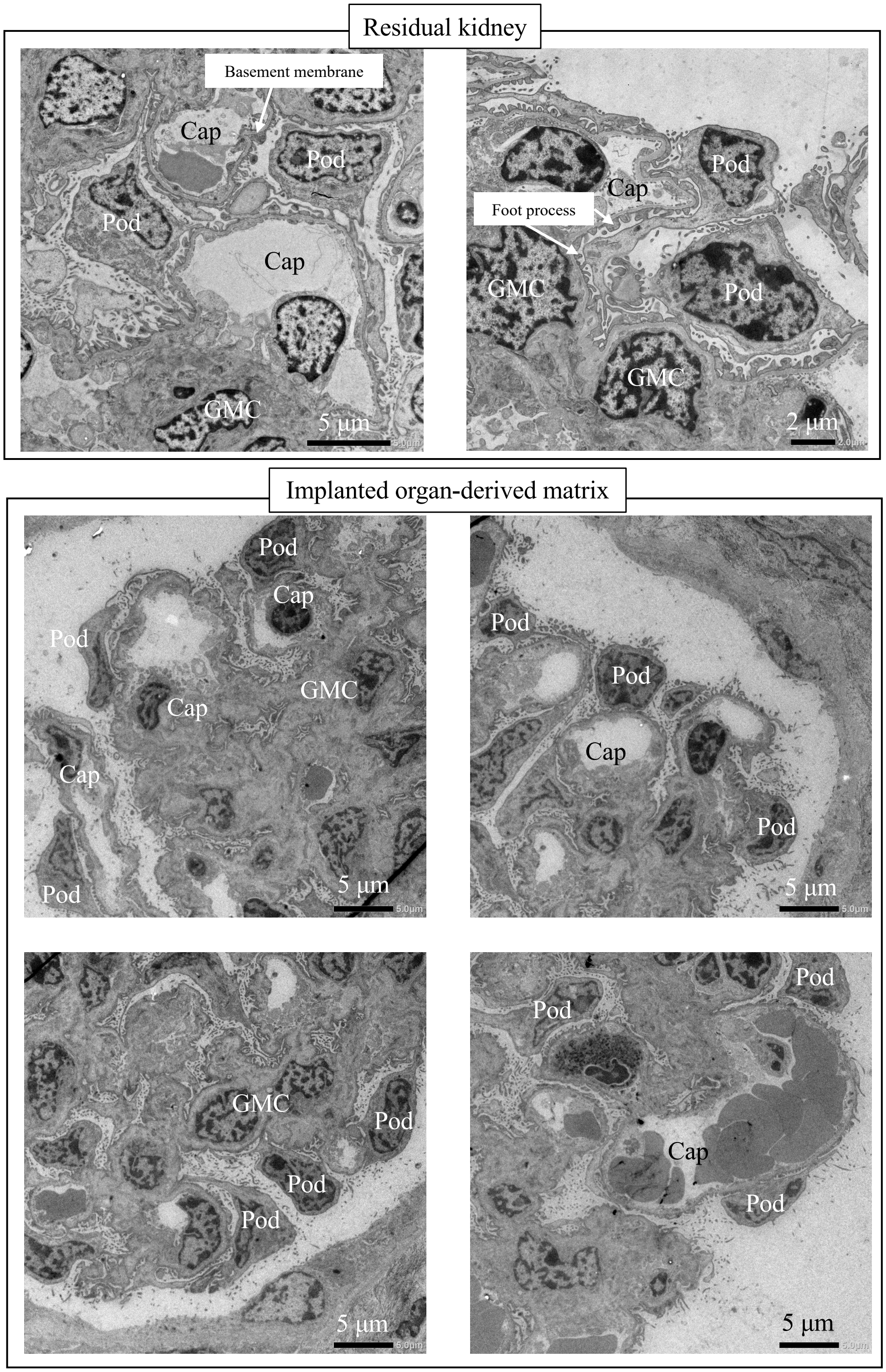

Supplementary Fig. 5 Formation of Pod, Cap and GMC in the glomeruli within the matrix.

TEM images of the glomeruli (top) within the residual kidney and (bottom) the matrix. Pod, podocyte; Cap, glomerular capillary; GMC, glomerular mesangial cell.

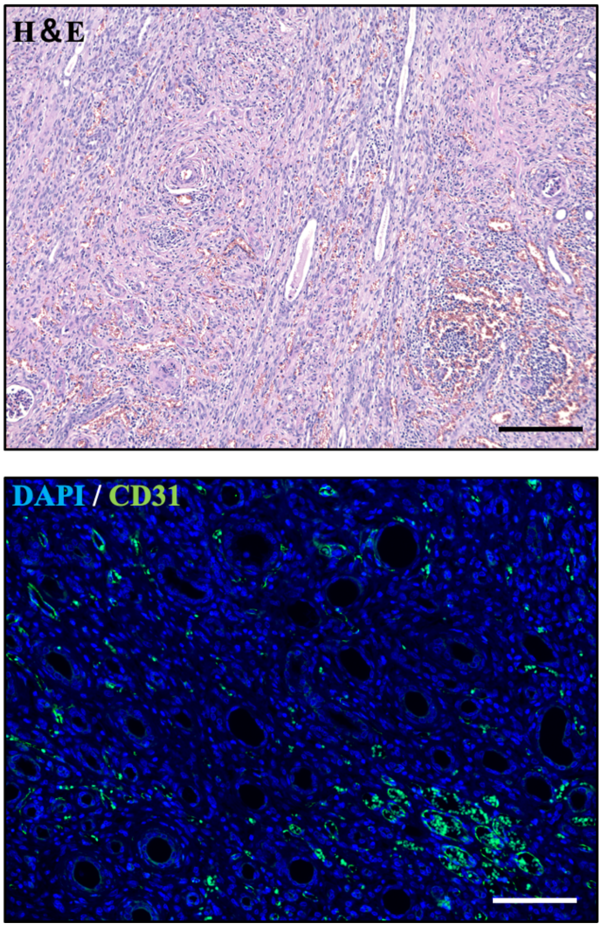

Supplementary Fig. 6 The expression of CD31 was negative around the resection area in the PN kidney.

(top) H&E staining near the resection line in the PN kidney and (bottom) CD 31 staining counterstained with DAPI. Scale bar: 30 μm for H&E, 100 μm for CD31.

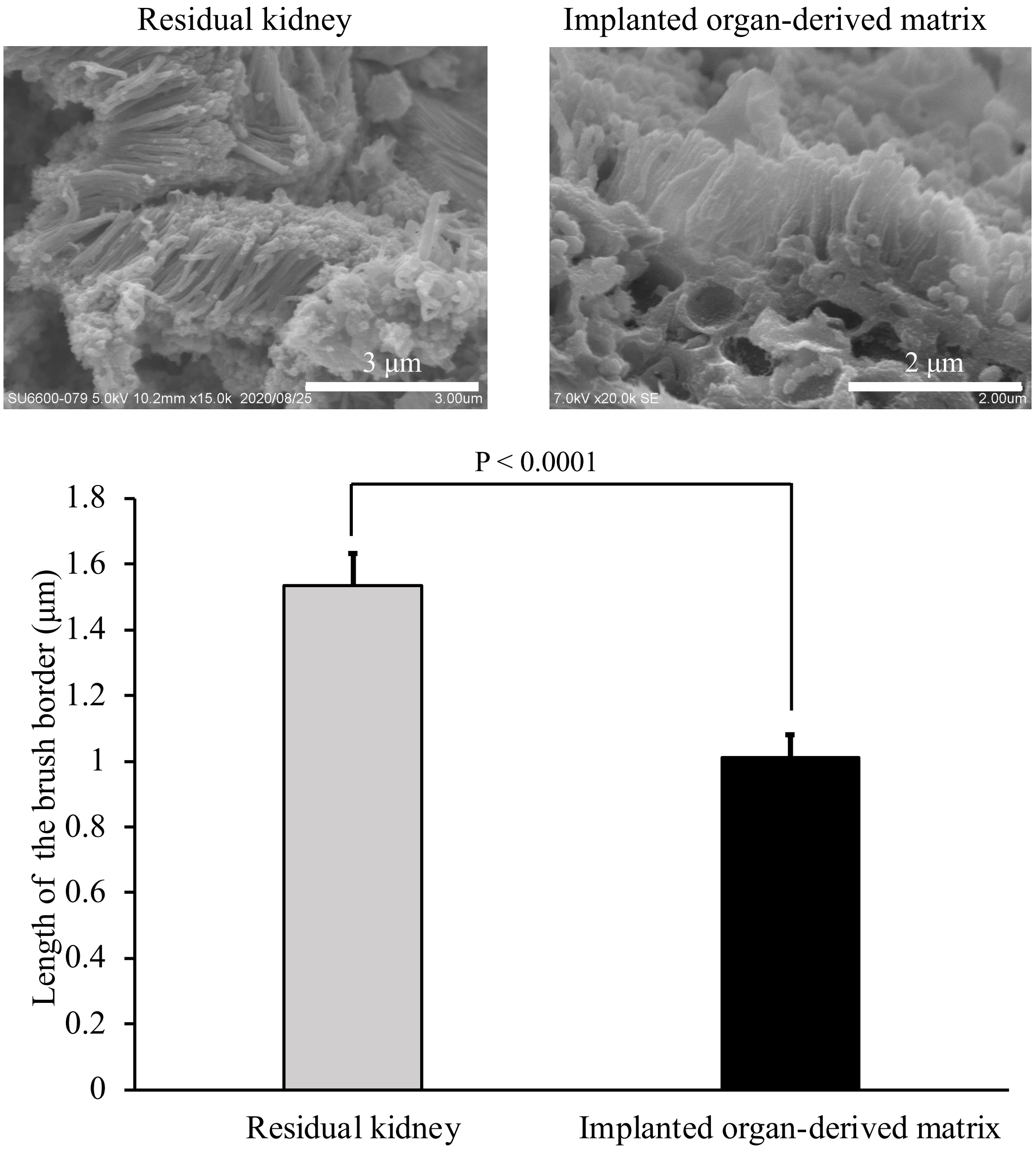

Supplementary Fig. 7 The brush borders in the matrix were shorter than those in the residual kidney.

(top) SEM images of the brush borders in the matrix and residual kidney and (bottom) the length of brush borders in each region. Bars represent the mean ± SD, (n = 10).

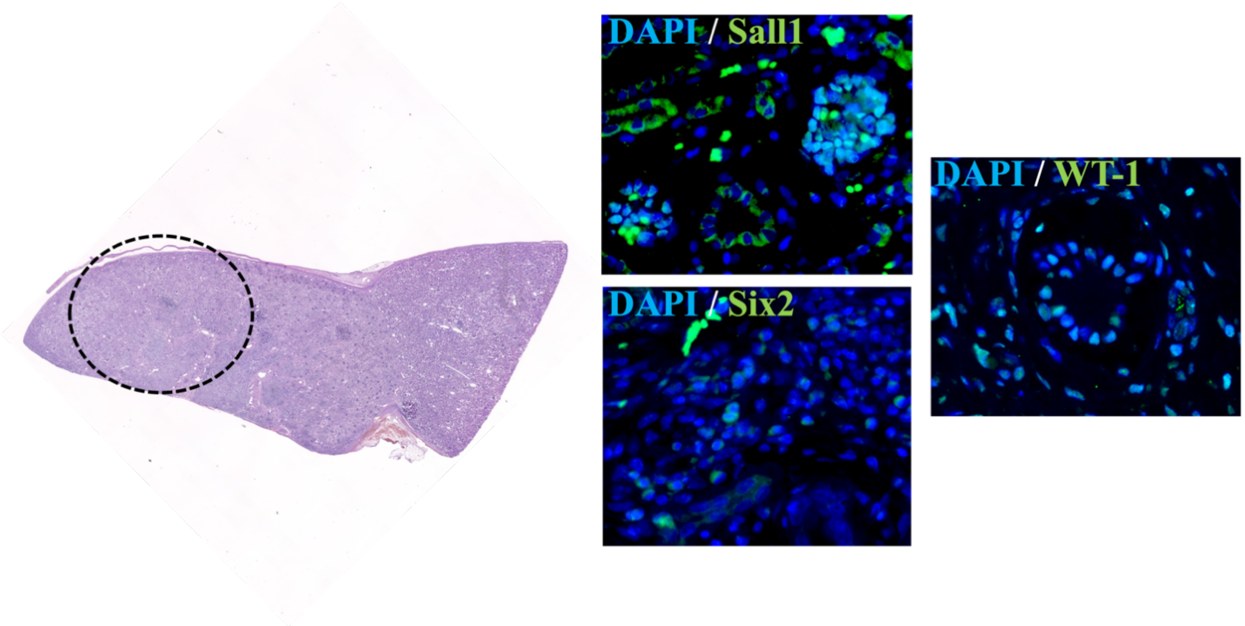

Supplementary Fig. 8 The progenitor markers are expressed in the regions away from the boundary of the matrix and residual kidney.

(leff) H&E montage image of the matrix sutured to the residual kidney. (right) Sall1, Six2, WT-1, and DAPI staining of cells in the implanted organ-derived matrix. The black-dotted circle in the H&E image shows the region where the expression of Sall1, Six2, and WT-1 was observed. The H&E and immunohistochemical images are the same as Fig. 4a and Fig. 5a, respectively.

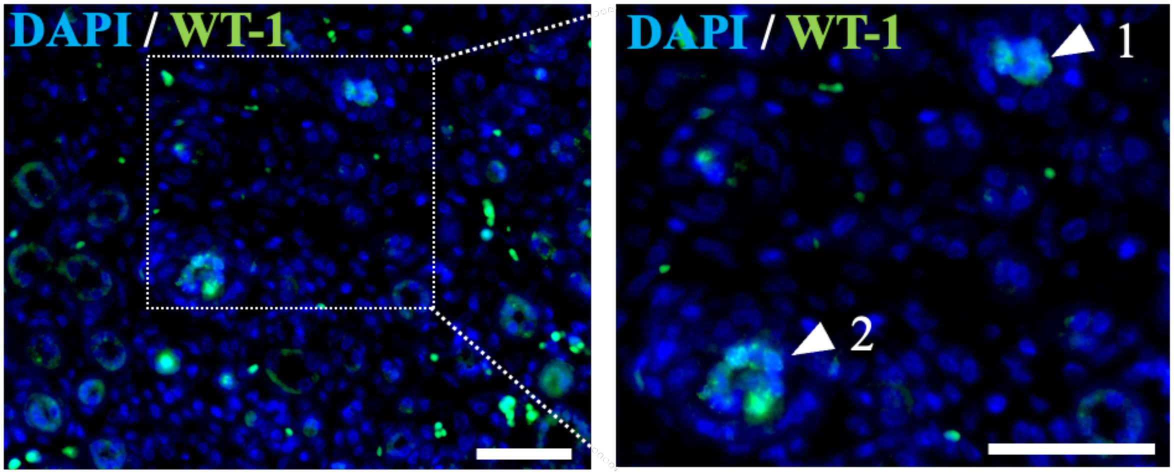

Supplementary Fig. 9 The cells forming the luminal structure inside the organ-derived matrix were positive for WT-1.

WT-1 and DAPI staining of cells in the matrix**.** The white arrowheads indicate the WT-1-positive cells in the luminal structures. Scale bar: 100 μm.

Supplementary Table. 1 Residual proteins in the organ-derived matrix: list 1 (No. 1–50)

Symbol	Description
COL6A3	Collagen alpha-3(VI) chain
HSPG2	basement membrane-specific heparan sulfate proteoglycan core protein isoform X17
HSPG2	basement membrane-specific heparan sulfate proteoglycan core protein isoform X16
PLEC	plectin isoform X4
COL12A1; LOC100156689	collagen alpha-1(XII) chain isoform X1
FLNA; LOC733585	filamin-A isoform X3
LAMA5	laminin subunit alpha-5 isoform X1
LOC100738123; COL1A1	Collagen alpha-1(I) chain isoform X1
COL1A2	Collagen alpha-2(I) chain precursor
LOC106508661; FREM2	FRAS1-related extracellular matrix protein 2
LOC106504153; AHNAK	neuroblast differentiation-associated protein AHNAK
COL6A5	Collagen alpha-5(VI) chain isoform X1
LOC396904; MYH9	myosin-9
TLN1	Talin-1 isoform x4
LOC100627924; MYH11	myosin-11
FN1	fibronectin
DYNC1H1	cytoplasmic dynein 1 heavy chain 1
SPTBN1	spectrin beta chain, non-erythrocytic 1 isoform X1
FLNC	filamin-C isoform X3
LOC100514785; LAMC1	laminin subunit gamma-1 precursor
COL14A1	Collagen alpha-1(XIV) chain isoform X1
COL14A1	Collagen alpha-1(XIV) chain isoform X2
LAMB2	Laminin subunit beta-2
CLTC	clathrin heavy chain 1 isoform X1
LOC100049693; SPTAN1	spectrin alpha chain, non-erythrocytic 1
LOC100623720; COL6A1	Collagen alpha-1(VI) chain
COL4A2	Collagen alpha-2(IV) chain
LAMB1	Laminin subunit beta-1
COL7A1	Collagen alpha-1(VII) chain
COL6A2	Collagen alpha-2(VI) chain
UTRN; LOC100523292	utrophin isoform X1
LOC396903; MYH10	myosin-10
PC	pyruvate carboxylase, mitochondrial
COL5A2	Collagen alpha-2(V) chain precursor
ACTN1	Alpha-actinin-1
ALB	albumin, partial
TNC	tenascin isoform X1
LOC100154530; TGM2	Protein-glutamine gamma-glutamyltransferase 2
LOC100511351; NID1	nidogen-1 isoform X1
TGFBI	transforming growth factor-beta-induced protein ig-h3
TN-X; TNXB	tenascin-X isoform X9
LTBP1; LOC100627243	latent-transforming growth factor beta-binding protein 1
EMILIN1	EMILIN-1 isoform X1
LAMA4	Laminin subunit alpha-4
LOC100156744; DSP	desmoplakin isoform X1
LOC100522394; VIM	vimentin isoform X1
PCK2	Phosphoenolpyruvate carboxykinase [GTP], mitochondrial
LOC100627396; FGG	fibrinogen gamma chain isoform X1
MYO1C	unconventional myosin-Ic isoform X4
LMNA	Prelamin-A/C

Supplementary Table. 2 Residual proteins in the organ-derived matrix: list 2 (No. 51–100)

Symbol	Description
LMNA	RecName: Full=Prelamin-A/C; Contains: RecName: Full=Lamin-A/C; Flags: Precursor
LOC100517137; ALDH1A1	Retinal dehydrogenase 1
LOC100623833; SYNM	synemin
POSTN	periostin isoform 1 precursor
POSTN	periostin isoform X1
EHHADH	Peroxisomal bifunctional enzyme
COL5A1	Collagen alpha-1(V) chain precursor
LOC100515336; COL4A1	Collagen alpha-1(IV) chain
LOC100516352; TUBB4B	Tubulin beta-4B chain
LOC100517284; ACTN4	alpha-actinin-4 isoform X
TUBB2A; TUBB	tubulin beta chain
LOC100153159; TINAG; LOC102159928	tubulointerstitial nephritis antigen isoform X1
LOC100620724; COL4A6	Collagen alpha-6(IV) chain
LOC100621536; PYGB	Glycogen phosphorylase, brain form
LOC100624785	Tubulin beta-2A chain
DDX3X	ATP-dependent RNA helicase DDX3X isoform X1
DMGDH	dimethylglycine dehydrogenase, mitochondrial isoform X1
EZR; LOC100152815	Ezrin
FASN	Fatty acid synthase
SCP2	Non-specific lipid-transfer protein
TLN2	talin-2 isoform X1
ACTA2	Actin, aortic smooth muscle
LONP1	Lon protease homolog, mitochondrial
PRPH; FAIM2	peripherin isoform X1
TNS1	tensin-1
ASS1	Argininosuccinate synthase
ATP5B	ATP synthase subunit beta, mitochondrial
LOC100125553; LOC100135051; TUBA1A	tubulin alpha-1A chain isoform X1
LOC100515339; KRT19	Keratin, type I cytoskeletal 19
LOC100624675; COL18A1	Collagen alpha-1(XVIII) chain isoform X1
SARDH	sarcosine dehydrogenase, mitochondrial isoform X1
TUBA4A	Tubulin alpha-4A chain
ACY1	Aminoacylase-1
ACY1	aminoacylase I
AKAP12	A-kinase anchor protein 12
IDH2	Isocitrate dehydrogenase [NADP], mitochondrial
LOC100151886; DPYSL2	dihydropyrimidinase-related protein 2 isoform X1
LOC100514347; DDX17	Probable ATP-dependent RNA helicase DDX17, partial
COL2A1	Collagen alpha-1(II) chain
KRT8	Keratin, type II cytoskeletal 8
LOC100620404; HNRNPR	heterogeneous nuclear ribonucleoprotein R isoform X2
LOC100621504; COL4A3	Collagen alpha-3(IV) chain
LOC100737091; COL4A5	collagen alpha-5(IV) chain isoform X1
LOC396904; MYH9	non-muscle myosin heavy chain II A, partial
MTHFD1	C-1-tetrahydrofolate synthase, cytoplasmic isoform X2
SEC31A	protein transport protein Sec31A isoform X1
SELENBP1	selenium-binding protein 1 isoform X1
SORBS1	sorbin and SH3 domain-containing protein 1 isoform X16
ACSM2B; LOC100510957	acyl-coenzyme A synthetase ACSM2B, mitochondrial isoform X2
COL4A4	collagen alpha-4(IV) chain

Supplementary Table. 3 Genes analyzed in the kidneys: list 1

Symbol	Description
ACTA2	Actin, alpha 2, smooth muscle, aorta
ACTC1	Actin, alpha, cardiac muscle 1
AKT1	V-akt murine thymoma viral oncogene homolog 1
ANGPT1	Angiopoietin 1
CCL2	Chemokine (C-C motif) ligand 2
CTGF	Connective tissue growth factor
CD40LG	CD40 ligand
CDH1	Cadherin 1, type 1, E-cadherin (epithelial)
COL14A1	Collagen, type XIV, alpha 1
LOC397571	Collagen, type I, alpha 1
COL1A2	Collagen, type I, alpha 2
COL3A1	Collagen, type III, alpha 1
LOC397502	Collagen, type IV, alpha 3 (Goodpasture antigen)
COL5A2	Collagen, type V, alpha 2
COL5A3	Collagen, type V, alpha 3
CSF2	Colony stimulating factor 2 (granulocyte-macrophage)
CSF3	Colony stimulating factor 3 (granulocyte)
CTNNB1	Catenin (cadherin-associated protein), beta 1, 88kDa
CTSK	Cathepsin K
CXCL11	Chemokine (C-X-C motif) ligand 11
CXCL12	Chemokine (C-X-C motif) ligand 12
LOC396594	Growth-regulated protein homolog gamma
DCN	Decorin
EDN1	Endothelin 1
EGF	Epidermal growth factor
EGFR	Epidermal growth factor receptor
EPHB2	EPH receptor B2
LOC100153504	Coagulation factor XIII, A1 polypeptide
LOC100514666	Fibrinogen alpha chain
FGF2	Fibroblast growth factor 2 (basic)
FGF7	Fibroblast growth factor 7
HBEGF	Heparin-binding EGF-like growth factor
IFNG	Interferon-gamma
IGF1	Insulin-like growth factor 1 (somatomedin C)
IL10	Interleukin 10
IL1A	Interleukin 1, alpha
IL1B	Interleukin 1 beta
IL2	Interleukin 2
IL4	Interleukin 4
IL6ST	Interleukin 6 signal transducer (gp130, oncostatin M receptor)
ITGA2	Integrin, alpha 2 (CD49B, alpha 2 subunit of VLA-2 receptor)
LOC100517053	Integrin, alpha 3 (antigen CD49C, alpha 3 subunit of VLA-3 receptor)
ITGA4	Integrin, alpha 4 (antigen CD49D, alpha 4 subunit of VLA-4 receptor)
ITGA5	Integrin, alpha 5 (fibronectin receptor, alpha polypeptide)
ITGA6	Integrin, alpha 6
ITGAV	Integrin, alpha V (vitronectin receptor, alpha polypeptide, antigen CD51)

Supplementary Table. 4 Genes analyzed in the kidneys: list 2

Symbol	Description
ITGB1	Integrin, beta 1 (fibronectin receptor, beta polypeptide, antigen CD29, includes MDF2, MSK12)
ITGB3	Integrin, beta 3 (platelet glycoprotein IIIa, antigen CD61)
ITGB5	Integrin, beta 5
ITGB6	Integrin, beta 6
LOC100154047	Cathepsin G-like
PLAUR	Urokinase plasminogen activator surface receptor-like
FGF10	Fibroblast growth factor 10
LOC100627044	WNT1-inducible-signaling pathway protein 1-like
WNT5A	Wingless-type MMTV integration site family, member5A
F3	Tissue factor
MET	Met proto-oncogene (hepatocyte growth factor receptor)
MIF	Macrophage migration inhibitory factor (glycosylation-inhibiting factor)
MMP1	Matrix metallopeptidase 1 (interstitial collagenase)
MMP2	Matrix metallopeptidase 2 (gelatinase A, 72dkDa gelatinase, 72kDa type IV collagenase)
MMP3	Matrix metallopeptidase 3 (stromelysin 1, progelatinase)
MMP7	Matrix metallopeptidase 7 (matrilysin, uterine)
MMP9	Matrix metallopeptidase 9 (gelatinase B, 92kDa gelatinase, 92kDa type IV collagenase)
MYLK	Myosin light chain kinase
PTGS2	Prostaglandin G/H synthase-2
PLAT	Plasminogen activator, tissue
PLAU	Plasminogen activator, urokinase
PLG	Plasminogen
PTEN	Phosphatase and tensin homolog
PTGS1	Prostaglandin-endoperoxide synthase 1 (prostaglandin G/H synthase and cyclooxygenase)
RAC1	Rac family small GTPase 1
SERPINE1	Serpin peptidase inhibitor, clade E (nexin, plasminogen activator inhibitor type 1), member 1
TAGLN	Transgelin
SMAD3	SMAD family member 3
STAT3	Signal transducer and activator of transcription 3 (acute-phase response factor)
TGFA	Transforming growth factor, alpha
TGFB1	Transforming growth factor, beta 1
TGFB2	Transforming growth factor, beta 2
TGFB3	Transforming growth factor, beta 3
TGFBR3	Transforming growth factor, beta receptor III
TNC	Tenascin C
TNF	Tumor necrosis factor
VEGFA	Vascular endothelial growth factor A
VTN	Vitronectin
ACTG1	Actin gamma 1
B2M	Beta-2-microglobulin
GAPDH	Glyceraldehyde-3-phosphate dehydrogenase
HPRT1	Hypoxanthine phosphoribosyltransferase 1
RPL13A	Ribosomal protein L13a
SGDC	Pig Genomic DNA Contamination
RTC	Reverse Transcription Control
PPC	Positive PCR Control
